# The role of miRNAs 34a, 146a, 320a and 542 in the synergistic anticancer effects of methyl 2-(5-fluoro-2-hydroxyphenyl)-1H- benzo[d]imidazole-5-carboxylate (MBIC) with doxorubicin in breast cancer cells

**DOI:** 10.7717/peerj.5577

**Published:** 2018-09-17

**Authors:** Mohadeseh Hasanpourghadi, Nazia Abdul Majid, Mohd Rais Mustafa

**Affiliations:** 1 Department of Pharmacology, Faculty of Medicine, University of Malaya, Kuala Lumpur, Malaysia; 2 Institute of Biological Sciences, Faculty of Science, University of Malaya, Kuala Lumpur, Malaysia

**Keywords:** Breast cancer, microRNA, Survivin, Synergism, NF-κB

## Abstract

Combination Index (CI) analysis suggested that MBIC and doxorubicin synergistically inhibited up to 97% of cell proliferation in ER^+^/PR^+^MCF-7 and triple negative MDA-MB-231 breast cancer cell lines. Moreover, treatment of the breast cancer cells with the combined drugs resulted in lower IC_50_ values in contrast to the individual drug treatment. Small noncoding microRNAs (miRNA) may function as non-mutational gene regulators at post-transcriptional level of protein synthesis. In the present study, the effect of the combined treatment of MBIC and doxorubicin on the expression level of several miRNAs including miR-34a, miR-146a, miR-320a and miR-542 were evaluated in MCF-7 and MDA-MB-231 breast cancer cell lines. These miRNAs have the potential to alter the protein level of survivin, the anti-apoptotic protein and reduce the metastatic activity in human breast cancer cell lines by interfering with the nuclear accumulation of NF-κB. Our results demonstrated the several fold changes in expression of miRNAs, which is drug and cell line dependent. This finding demonstrated a functional synergistic network between miR-34a, miR-320a and miR-542 that are negatively involved in post-transcriptional regulation of survivin in MCF-7 cells. While in MDA-MB-231 cells, changes in expression level of miR-146a was correlated with inhibition of the nuclear translocation of NF-κB. The overall result suggested that alteration in protein level and location of survivin and NF-κB by miR-34a, miR-320a, miR-146a and miR-542, remarkably influenced the synergistic enhancement of combined MBIC and doxorubicin in treatment of aggressive and less aggressive human breast cancer cell lines.

## Introduction

Evaluation of drug-drug synergistic interactions are important in medicine (Zhao, Au & Wientjes, 2010). Synergism is one of the nature of interaction, when the overall effect of two drugs in combination is higher than the effect of each individual drug alone. Synergism is attributed to multiplicity of intracellular targets of the individual drugs and their interactions (Chou, 2006). We recently reported that doxorubicin, a well-known DNA-damaging agent (DDA) (Gavet & Pines, 2010) and MBIC, a recently introduced Microtubule Targeting Agent (MTA) (Hasanpourghadi, Pandurangan & Mustafa, 2017) in combination caused a further reduction of 39.5% and 56.8% of tumor volume compared to doxorubicin or MBIC monotherapy respectively (Hasanpourghadi et al., 2017).

Recently accumulating evidence indicate the alteration of particular miRNAs are involved in the initiation and development of carcinogenesis. Similarly, the expression profiling of selective miRNAs is associated with the sensitivity of the cancer cells to the anticancer drugs. More specifically, miRNAs are modulating the sensitivity of the cancer cells to anticancer drugs (Blower et al., 2008). miRNAs are small noncoding RNAs that control the formation, stability and function of messenger RNA post-transcriptionally (Shi et al., 2014). Growing evidence reveals that miRNAs contribute to the efficacy of drugs by altering the expression of proteins that are targeted by those particular drugs (Rodrigues et al., 2011; Pogribny & Beland, 2013; Choudhuri, Cui & Klaassen, 2010). Hence, expanding the knowledge about those miRNAs involved in the expression of proteins that are targets of respective drugs is becoming increasingly important (Zheng et al., 2010). miRNA-based processes offer a valuable tool for understanding the synergistic outcome of drug combinations (Richner et al., 2015). Previously, the importance of the bi-functional role of survivin protein was reported, in order to increase the benefits that a patient with breast cancer may receive from anticancer effect of MBIC (Hasanpourghadi et al., 2017). Therefore, in the present study we examined the association of several miRNAs that are reported to be involved in the expression of survivin protein following treatment with anticancer drugs. miR-34a, miR-320a and miR-542 are reported to target mRNA transcripts of the anti-apoptotic protein survivin (Cao et al., 2013; Diakos et al., 2010; Yoon et al., 2010).

MCF-7 and MDA-MB-231 are metastatic breast cancer cells with different level of aggression. This common characteristic of two cell lines motivated us to add a miRNA to the study, which is known to correlate with the metastasis mechanism. miR-146a is reported to reduce the metastatic activity of MDA-MB-231 cells (Bhaumik et al., 2008) through inhibition of the activity of NF-κB (Bhaumik et al., 2008). In this study, the cytotoxic effects of MBIC in the presence of several chemotherapeutic drugs was examined. The combination of doxorubicin with MBIC demonstrated the greatest synergistic induction of cell death in breast cancer cell lines. Secondly, the effect of the drug combination on the expression level of miR-34a, miR-146a, miR-320a and miR-542 was evaluated. Moreover, the potential contribution of miRNAs in the protein level and activation of two target-proteins, survivin and NF-κB were evaluated.

## Material & Methods

### Culture condition

Aggressive and highly metastatic human breast cancer cell line, MDA-MB-231 and less aggressive human breast cancer cell line, MCF-7 were obtained from American Type Culture Collection (ATCC, Manassas, VA, USA). Cells were maintained in RPMI-1640 medium and supplemented with 1% penicillin/streptomycin and 10% Fetal Bovine Serum (FBS) obtained from Gibco, Thermo Fisher Scientific (Rockville, MD, USA).

### Drug combination treatment

To assess the possible synergistic effect of MBIC with conventional drugs, cells were seeded and treated, then the half-maximal inhibitory concentration (IC_50_) of drugs were calculated in single-drug or multiple-drug treatments, and finally calculated the Combination Index (CI) value. Cells were acquired in 1 × 10^4^ cells/well and were incubated at 37 °C overnight. Thereafter, drugs were applied into the cells. First, the IC_50_ value of MBIC and selected conventional anticancer drugs—colchicine, paclitaxel, nocodazole, tamoxifen, 5-fluorouracil (5-FU) and doxorubicin were determined in single-drug treatment. The IC_50_ values of MBIC in both breast cancer cell lines, were reported in our previous study (Hasanpourghadi et al., 2017). IC_50_ value represents the inhibitory concentration of the drug against the cell, and is used to evaluate the performance of the drug in terms of the best efficacy. There are millions of compounds introduced as anticancer drug, which first must pass the IC_50_ value test in order to be classified as drugs with most likely desired qualities. Further, the best lead compounds are tested in different concentrations to be evaluated for different properties in different level of toxicities, such as highest toxicity with lowest possible concentration and/or best effectiveness and advantages with a balanced dosage (Sebaugh, 2011).

Treatments of combined drugs were then evaluated in three types of combination setting. In the first setting, MCF-7 and MDA-MB-231 cells were incubated with various concentrations of MBIC and various concentrations of the second drug (one conventional drug per plate). Unlike the first setting, in second and third settings, only the concentration of one of two drugs are variable. The second combination setting was designed to determine the combination effects of each conventional drug at various concentrations, while the concentration of MBIC remained fixed at IC_50_ concentration. Third combination setting was designed to investigate combination effects of MBIC at various concentrations, while the concentration of each conventional drug remained fixed at their IC_50_ concentration (according to the previously reported method (Tsakalozou, Eckman & Bae, 2012)). The variable concentrations of MBIC used in this study were between 0.048 µM to 100 µM.

Moreover, in combination study, obtaining the synergism, additivity and antagonism are required by calculating the CI value. MTT assay was done 24 h after treatment and absorbance was quantified to determine the IC_50_ values. CI value was determined using the following equation:

\begin{eqnarray*}\text{CI}~(50\text{%})=[\text{MBIC}~\text{conc}]/[\text{MBIC}~\text{conc}~\text{X}]+[\text{2nd}~\text{drug}~\text{conc}]/[\text{2nd}~\text{drug}~\text{conc}~\text{X}]+\nonumber\\\displaystyle [\text{MBIC}~\text{conc}]\times [\text{2nd}~\text{drug}~\text{conc}]/[\text{MBIC}~\text{conc}~\text{X}]\times [\text{2nd}~\text{drug}~\text{conc}~\text{X}]. \end{eqnarray*}

“X” is concentration of MBIC or second drug (one of conventional anticancer drugs) alone wherein they caused 50% inhibition. The concentration of MBIC or second drug without (X), is concentration of each in combination, wherein together they caused 50% inhibition (Tsakalozou, Eckman & Bae, 2012). Moreover, 75%, 90%, 95% and 97% inhibition of cell proliferation were obtained as well.

### MicroRNA assay

#### Quantitative Real-Time PCR primer design

Five miRNAs were selected, and miRbase (http://www.mirbase.org) was used to design the primers. The data such as sequences of mature miRNA, accession of sequences of stem-loop were acquired from this database, and primers were ordered from Ribobio Co., LTD (Guangzhou, China). Individual miRNA profiling was analyzed by qRT-PCR, to ensure accurate miRNA quantification in qRT-PCR study where highly conserved U6 snRNA was selected as normalizer/endogenous reference. The sequences of mature miRNA and the accession code are provided in Table 1. The reverse primer sequence used in this study was the universal miR-Reverse Primer (Code # ssD089261711).

**Table 1:** Acquired primers for PCR analysis of microRNAs. The sequences of mature miRNA and the accession code of stem-loops are provided. The selection of 3′ (3p) or 5′ (5p) arm is based on which arm is more dominant. The dominant arms are selected over passenger arms.

MicroRNA	Mature microRNA	Accession code
hsa^a^-miR-34a-5p	hsa-miR-34a-5p MIMAT0000255 UGGCAGUGUCUUAGCUGGUUGU	MI0000268
hsa-miR-146a-5p	hsa-miR-146a-5p MIMAT0000449 UGAGAACUGAAUUCCAUGGGUU	MI0000477
hsa-miR-320a-3p	hsa-miR-320a MIMAT0000510 AAAAGCUGGGUUGAGAGGGCGA	MI0000542
hsa-miR-542-3p	hsa-miR-542-3p MIMAT0003340 UCGGGGAUCAUCAUGUCACGAGA	MI0003686

**Notes.**

a “hsa” refers to human microRNA.

Source: http://www.mirbase.org.

#### Extraction of total RNA

Given to the results of combination therapy, as the best synergistic effect was observed in treatment of breast cancer cell lines with MBIC and doxorubicin, next the expression of few miRNAs under effect of combination of these two drugs were evaluated. MDA-MB-231 and MCF-7 cells were seeded in flask, and at 80% of confluence, the cells were treated with either MBIC or doxorubicin in single-drug or multiple-drug treatment settings for 24 h. MCF-7 and MDA-MB-231 cell lines treated with MBIC, doxorubicin or combination of these two drugs, were divided into four groups. MBIC-treated group included untreated, treated at $ \frac{1}{2} $ × IC_50_ concentration (0.36 µM for MCF-7; 10.2 µM for MDA-MB-231), at IC_50_ concentration (0.73 µM for MCF-7; 20.4 µM for MDA-MB-231), and at 2 × IC_50_ concentration (1.5 µM for MCF-7; 40 µM for MDA-MB-231). Doxorubicin treatment group included untreated, doxorubicin-treated at $ \frac{1}{2} $ × IC_50_ concentration (2.79 µM for MCF-7; 4.87 µM for MDA-MB-231), at IC_50_ concentration (5.58 µM for MCF-7; 9.75 µM for MDA-MB-231), and at 2 × IC_50_ concentration (11.16 µM for MCF-7; 19.5 µM for MDA-MB-231). Combination treatment groups were treated with the concentration of MBIC and doxorubicin wherein together they caused 50% of cell death (Tables 2 & 3).

**Table 2:** Combination effect of conventional drugs with MBIC in treatment of MCF-7 cells. Combination Index (CI) algorithm was used to quantitatively determine the type of interactions for conventional anticancer drug combinations with MBIC in treatment of MCF-7 human breast cancer cells. Synergism is shown in green if CI is smaller than 1 (CI < 1); antagonism is shown in purple if CI is above 1 (CI > 1).

Conventional anticancer Drugs	IC_50_ (µM) MCF-7				CI value at inhibition of				
	Monotherapy		In combination						
	Drug 1	MBIC	Drug 1	MBIC	50%	75%	90%	95%	97%
Colchicine	3.28 ± 0.11	0.73 ± 0.06	1.05 ± 0.07	0.11 ± 0.05	0.88	0.82	0.74	0.52	0.35
Nocodazole	5.12 ± 0.07		1.84 ± 0.28	0.25 ± 0.07	0.74	0.94	0.84	0.71	0.56
Paclitaxel	0.01 ± 0.0002		0.007 ± 0.0001	0.65 ± 0.005	0.57	0.51	0.29	0.15	0.09
Doxorubicin	5.58 ± 1.02		0.78 ± 0.92	0.12 ± 0.02	0.89	0.70	0.48	0.37	0.23
Tamoxifen	10.78 ± 0.69		4.07 ± 1.03	0.44 ± 0.05	1.40	1.23	0.81	0.67	0.46
5-FU	9.71 ± 0.62		2.91 ± 0.03	0.31 ± 0.01	1.05	0.96	0.65	0.30	0.26

**Table 3:** Combination effect of conventional anticancer drugs with MBIC in treatment of MDA-MB-231 cells. Combination Index (CI) algorithm was used to quantitatively determine the type of interactions for conventional anticancer drug combinations with MBIC in treatment of MDA- MB-231 human breast cancer cells. Synergism is shown in green if CI is smaller than 1 (CI < 1); antagonism is shown in purple if CI is above 1 (CI > 1).

Conventional anticancer drugs	Combination ratio IC_50_ (µM) MDA-MB-231				CI value at inhibition of				
	Monotherapy		In combination						
	Drug 1	MBIC	Drug 1	MBIC	50%	75%	90%	95%	97%
Colchicine	8.79 ± 0.23	20.42 ± 0.23	3.32 ± 0.51	9.23 ± 1.09	1.08	1.07	1.01	0.96	0.91
Nocodazole	10.31 ± 0.19		4.72 ± 0.98	10.02 ± 1.09	2.20	2.06	1.99	1.96	1.90
Paclitaxel	0.026 ± 0.002		0.001 ± 0.0002	6.08 ± 0.39	1.05	0.99	0.95	0.91	0.87
Doxorubicin	9.75 ± 0.67		1.78 ± 0.11	4.01 ± 0.25	0.94	0.88	0.79	0.64	0.52
Tamoxifen	23.61 ± 0.69		14.60 ± 2.18	10.54 ± 0.97	1.92	1.88	1.84	1.80	1.76
5-FU	21.07 ± 0.23		12.01 ± 1.64	10.76 ± 1.56	2.08	1.50	1.10	0.92	0.72

All washing and water-dilution steps throughout this experiment, was done with DNase-RNase-free 1 ×PBS (Cat # 46-013-CM; Corning, Corning, NY, USA) and DNase-RNase-free molecular grade water (Cat # 46-000-CV; Corning, Corning, NY, USA), respectively. Total RNA was extracted by miRCURY™ RNA isolation kit (Cat # 300110; Exiqon, Vedbaek, Denmark) according to the manufacturer’s protocol. In brief, cells were lysed by directly adding 600 µl of lysis buffer supplied with 1% β-mercaptoethanol in the flask. Cells were detached and lysed by gently tapping the flask and swirling the buffer around the flask for 5 minutes. Next, 200 µl of 100% DNase-RNase-free molecular grade ethanol was added to the lysate prior to the extraction. In this step, cells were passed 10 times through a 25-gauge needle attached on a 1 ml syringe. The samples were washed and eluted according to the protocol, until purified RNA was obtained, snapped-freezed in liquid nitrogen and stored at −80 °C. Next, the concentration of RNA was determined using Nanodrop 2000C Spectrophotometer (Thermo Scientific, Waltham, MA, USA), and each template RNA was adjusted to 5 ng/µl by water-dilution prior to complementary DNA (cDNA) synthesis.

#### Reverse transcription

In order to generate the complementary DNA (cDNA) from template RNA, Reverse transcription (RT) reaction was prepared and set up using miRCURY™ Universal RT miRNA PCR, cDNA synthesis kit II (Cat # 203301; Exiqon, Vedbaek, Denmark) according to manufacturer’s protocol. Briefly, 10 µl of RT reaction was obtained for each sample and were incubated at 42 °C for 60 minutes, followed by heat-inactivation at 95 °C for 5 minutes, and cooled at 4 °C.

#### Amplification of Real-Time PCR reaction

Real-time PCR amplification was prepared and set up using miRCURY LNA™ miRNA PCR, ExiLENT SYBR^®^ Green master mix (Cat # 203403; Exiqon, Vedbaek, Denmark), according to manufacturer’s protocol in ice, protected from light. PCR master mix, specific primers and cDNA template were mixed corresponding to 10 µl of total real-time PCR reaction. Real-time PCR cycle condition was set up according to the manufacturer’s protocol. Briefly, PCR was performed for 10 minutes at 95 °C for polymerase denaturation, and 10 seconds at 95 °C and 1 minute at 60 °C for 45 amplification cycles, and finalized by a dissociation curve for 5 seconds per each 0.5 °C, using Applied Biosystems StepOnePlus™ system. The fold changes were calculated by the Livak method (ΔΔCT method) according to the following formula:

\begin{eqnarray*}& & \Delta \text{CT}=\text{Average CT of target miRNA}-\text{Average CT of reference RNA (U6)} \end{eqnarray*}

\begin{eqnarray*}& & \Delta \Delta \text{CT}=\Delta \text{CT of treated}-\Delta \text{CT of untreated} \end{eqnarray*}

\begin{eqnarray*}& & \text{Fold differences in target miRNA relative to untreated}={2}^{-\Delta \Delta \text{CT}}. \end{eqnarray*}

#### Western blot analysis: evaluating the survivin protein level

We were motivated to investigate the association of selected miRNAs, expression of survivin protein and their correlation with the effect of anticancer drugs. Therefore, next the protein level of survivin was evaluated after cells were incubated with MBIC, doxorubicin, and combination of both drugs for 24 hours. Cells were treated with MBIC, doxorubicin and their combination at 2× of IC_50_ concentration of each drug. The selected concentrations of MBIC and doxorubicin in combination therapy was twice of sufficient concentration that caused 50% of cell death. Cells were seeded, treated, lysed and loaded for western blot analysis as described previously (Hasanpourghadi et al., 2016). The primary antibody used to probe target protein was anti-survivin (16 kDa; 1:1,000 µl) (CST, Framingham, MA, USA), and mouse anti-β-actin (42 kDa; 1:40,000 µl) antibodies. The concentration equal of 2 × IC_50_ value was selected to be consistent with the result of miRNAs at their highest or lowest expression levels.

#### Cytosol/nuclear extraction

The translocation of several proteins into the nucleus is the key mechanism of certain cellular activities, as the initiation of some cellular activities requires this translocation, internal activation of protein and cooperation with endogenous nuclear proteins. In this part of study, detection of translocation of NF-κB from cytosol inside the nucleus with or without drug treatment was required. To find the connection between drug-induced different expression level of miR-146a and activation of NF-κB in MDA-MB-231 and MCF-7 cell lines, we investigated whether NF-κB is translocated into nucleus from the cytosol under effect of drugs. In this experiment, 10 ng/ml lipopolysaccharide (LPS) was used in positive control group. Each cell lines were divided into five groups of treatment, including untreated, LPS-treated, MBIC-treated, doxorubicin-treated and combination-treated groups.

To prepare the cytosol/nuclear extract, 5 ×10^6^ MDA-MB-231 and MCF-7 cell line were seeded and harvested 24 hours after treatment. Cells were washed with 1× PBS and were processed for cytoplasmic and nuclear protein fractions using NE-PER nuclear/cytoplasmic extraction reagent kit (Cat # 78833; Thermo Scientific, Waltham, MA, USA) according to the manufacturer. In brief, harvested cells were centrifuged at 500× g for 5 minutes. Cell pellets were treated with 500 µl of Cytoplasmic Extraction Reagent 1 (CERI), and were vortexed for 15 s on the highest setting to totally suspend the cell pellet. Tubes were incubated on ice for 10 minutes and then 27.5 µl of CERII was added to each tube. Tubes were vortexed for 5 seconds on the highest setting and followed by incubation in the ice for 1 minute. Tubes were centrifuged at 16,000× g for 5 minutes. After centrifugation, the supernatant that is the cytoplasmic extract, was transferred into a pre-chilled tube. The insoluble part of fraction contains nuclei proteins, were treated with 250 µl of Nuclear Extraction Reagent (NER). Tubes were kept on ice and were vortexed for 15 seconds every 10 minutes for total of 40 minutes. Tubes were centrifuged at 16,000× g for 10 minutes and the supernatant which contains nuclear extract fraction, was immediately transferred to a pre-chilled tube.

#### Western blot analysis: evaluating activation of NF-κB

The extracted nuclear and cytoplasmic proteins were used for western blot analysis. GAPDH and Lamin B1 proteins were used as markers of cytoplasm and nucleus respectively. These two endogenous markers were also probed as negative controls for the opposing fractions. GAPDH was used as negative control for nuclear fraction, while Lamin B1 was used as negative control for the cytosolic fraction. The primary antibody used to probe target proteins were anti-NF-κB (65 kDa; 1:1,000 µl) (CST, Framingham, MA, USA), anti-GAPDH (35 kDa; 1:20,000 µl) (Santa Cruz Biotechnology, Santa Cruz, CA , USA), and mouse anti-Lamin B1 (66 kDa; 1:20,000 µl) (CST, Framingham, MA, USA) antibodies.

#### Statistical analysis

A one-way analysis of variance (ANOVA) at statistically significance levels that were expressed as *P* value ≤ 0.05 shown as “*”; *P* value ≤ 0.01 shown as “**”; *P* value ≤ 0.001 shown as “***”; *P* value ≤ 0.0001 shown as “****” were conducted. *P* value > 0.05 was considered not significant and was shown as “ns”. The Bonferroni pos *t*-test was used to test the statistical differences between control and treated groups. Statistical analysis was performed using GraphPad Prism version 7.00 (Graph Pad Software, San Diego, CA, USA). The intensities of western blot’s protein bands were quantified by imageJ version 1.51j8 (NIH, Bethesda, MD, USA), by basic intensity quantification. Data were expressed as mean ± SD of three independent experiments.

## Results

### MBIC displayed a synergistic effect with doxorubicin in MCF-7 and MDA-MB-231 cell lines

To maximize the cytotoxic effect of MBIC, breast cancer cells were sequentially treated with different known anticancer drugs and IC_50_s were determined. In Tables 2 and  3, a Combination Index (CI) algorithm was used to quantitatively determine the type of interactions for each drug combination as follows, synergism if CI is smaller than 1 (CI < 1), additivity if CI is equal 1 (CI = 1), and antagonism if CI is above 1 (CI > 1). Tables 2 and 3 showed the results following combination of MBIC with each of the six conventional anticancer drugs in MCF-7 and MDA-MB-231 cell lines. The synergistic effects of combination of two drugs are shown in green. This color represented two drugs that in combination have higher effect than the effect of each individual drug. The antagonistic effect where two drugs in combination that have less effect compared to each individual drug, was shown in purple in Tables 2 and  3. Besides, the synergistic and antagonistic effects were classified based on the percentage of cells killed by the combined drugs (50% to 97% of cell death). Doxorubicin exhibited synergistic effect with MBIC at throughout the entire range of 50% to 97% of inhibition in both MCF-7 and MDA-MB-231 cell lines. Another interesting point was that the concentration of either MBIC or doxorubicin in combination that is required for killing 50% of the cells, decreased significantly, especially in MCF-7 cells. Similarly, colchicine, nocodazole and paclitaxel exhibited synergistic effects with MBIC at the full range of 50% to 97% in MCF-7 but not in MDA-MB-231 cell line (Tables 2 & 3). Nocodazole and tamoxifen demonstrated additive effects for the entire scopes of CI value (50% to 97%) in MDA-MB-231 cells. However, colchicine, paclitaxel and 5-FU in combination with MBIC, indicated selective synergistic effect ranging between 50% to 97% of inhibition, in both breast cancer cell lines. In Tables 2 and  3 synergism is displayed in green, while antagonism is exhibited in purple.

### MicroRNA profiling

**Figure 1:**
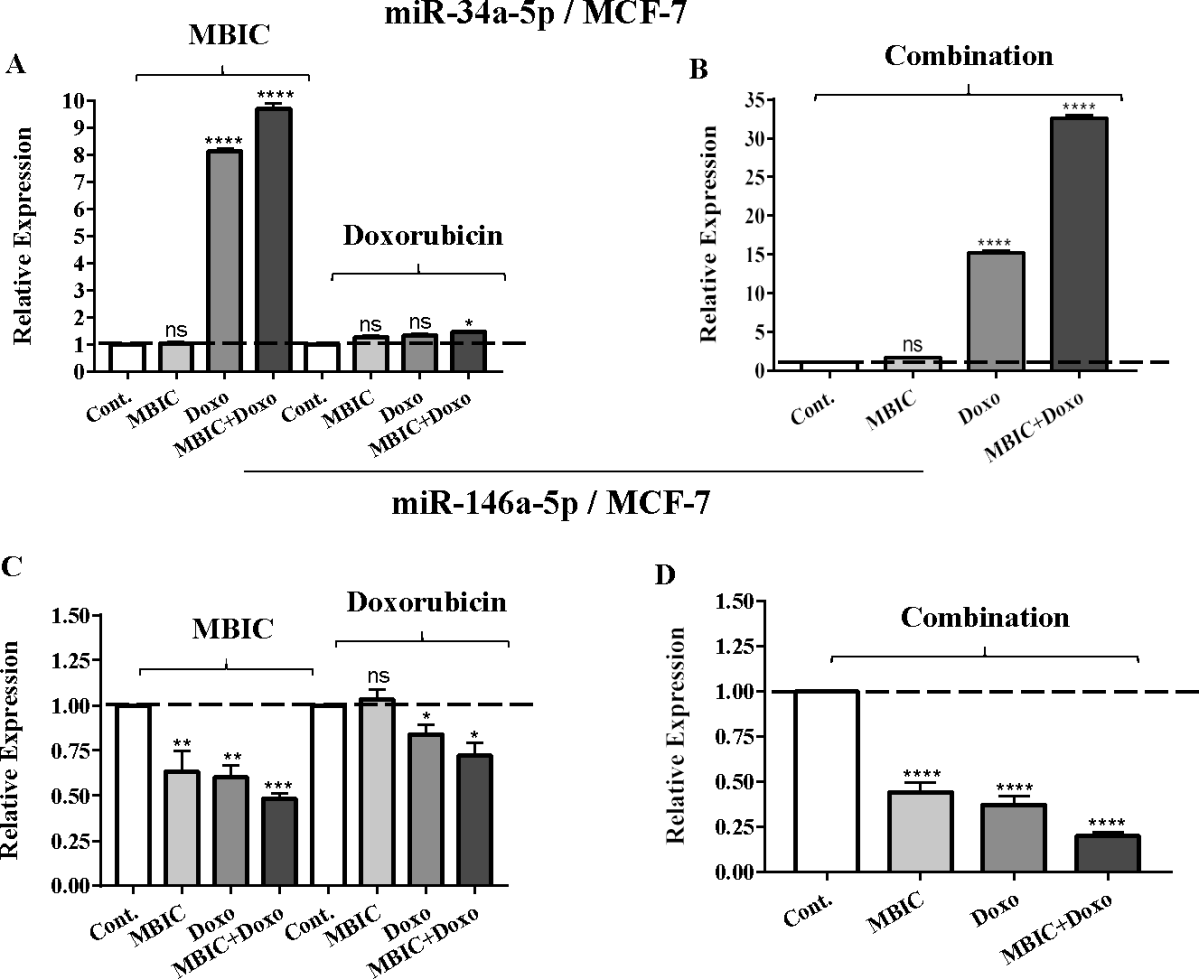
The role of miRNAs (miRs) miR-34a and miR-146a in synergistic effect of MBIC with doxorubicin in MCF-7 cells. Total RNA was extracted and reverse transcribed with their specific designed primers. The level of miRs were normalized to a small RNA (U6) and compared with this endogenous control (set as one-fold). Individual miRNA profiling was done using qRT-PCR analysis. The expression level of miR-34a (A & B) and miR-146a (C & D) in MCF-7 cells following 24 h treatment with MBIC, doxorubicin (A & C) and combination (B & D) of these two drugs are shown. The bars show the fold change of each treated group compared with untreated group (Cont.) as a horizontal dashed line. * *p* < 0.05. ** *p* < 0.01, *** *p* < 0.001, **** *p* < 0.0001 and “ns” indicates not significant compared with untreated control (Cont.). Results are mean + standard deviation of three independent experiments.

**Figure 2:**
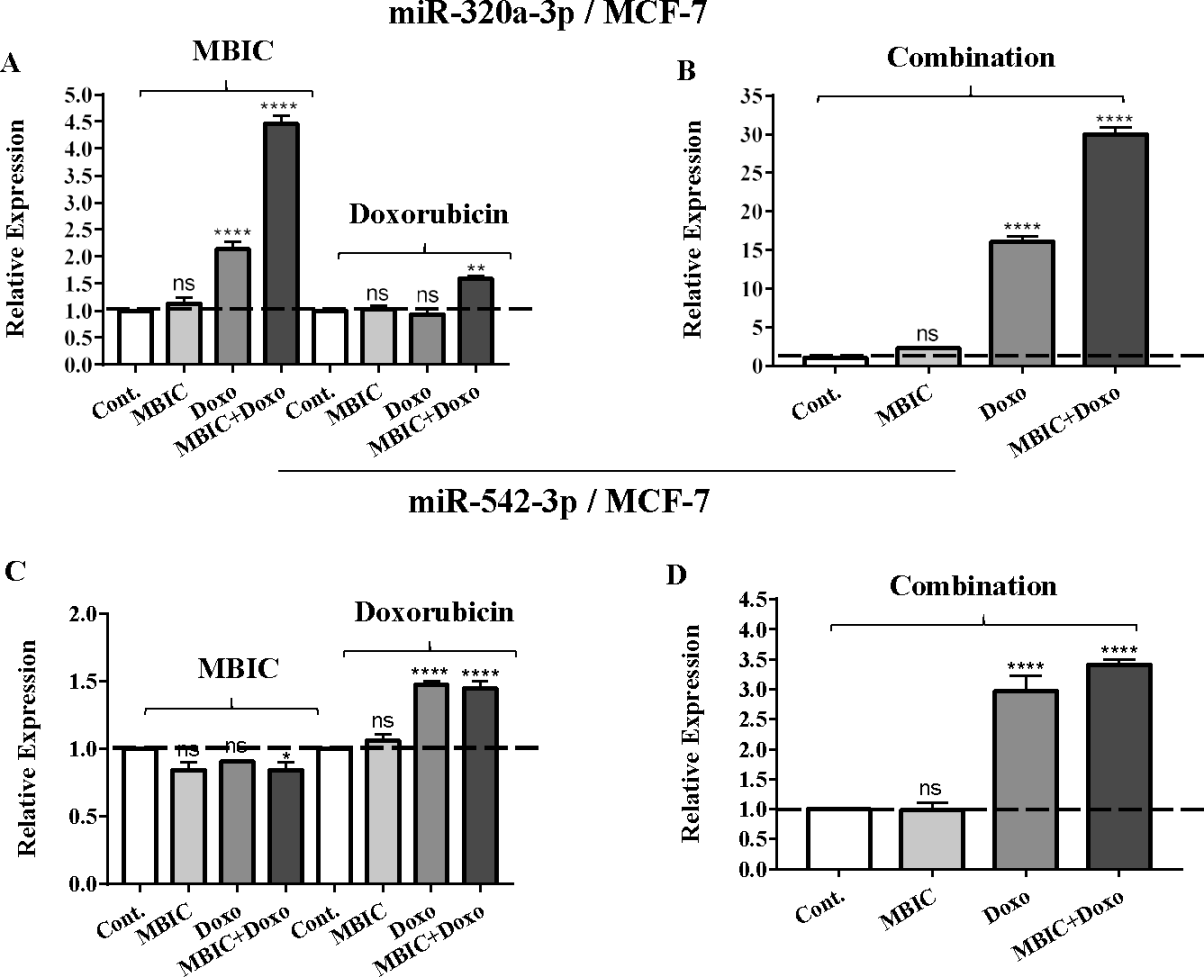
The role of miRNAs (miRs) miR-320a and miR-542 in synergistic effect of MBIC with doxorubicin in MCF-7 cells. Total RNA was extracted and reverse transcribed with their specific designed primers. The level of miRs were normalized to a small RNA (U6) and compared with this endogenous control (set as one-fold). Individual miRNA profiling was done using qRT-PCR analysis. The expression level of miR-320a (A & B) and miR-542a (C & D) in MCF-7 cells following 24 h treatment with MBIC, doxorubicin (A & C) and combination (B & D) of these two drugs are shown. The bars show the fold change of each treated group compared with untreated group (Cont.) as a horizontal dashed line. * *p* < 0.05. ** *p* < 0.01, **** *p* < 0.0001 and “ns” indicates not significant compared with untreated control (Cont.). Results are mean + standard deviation of three independent experiments.

**Figure 3:**
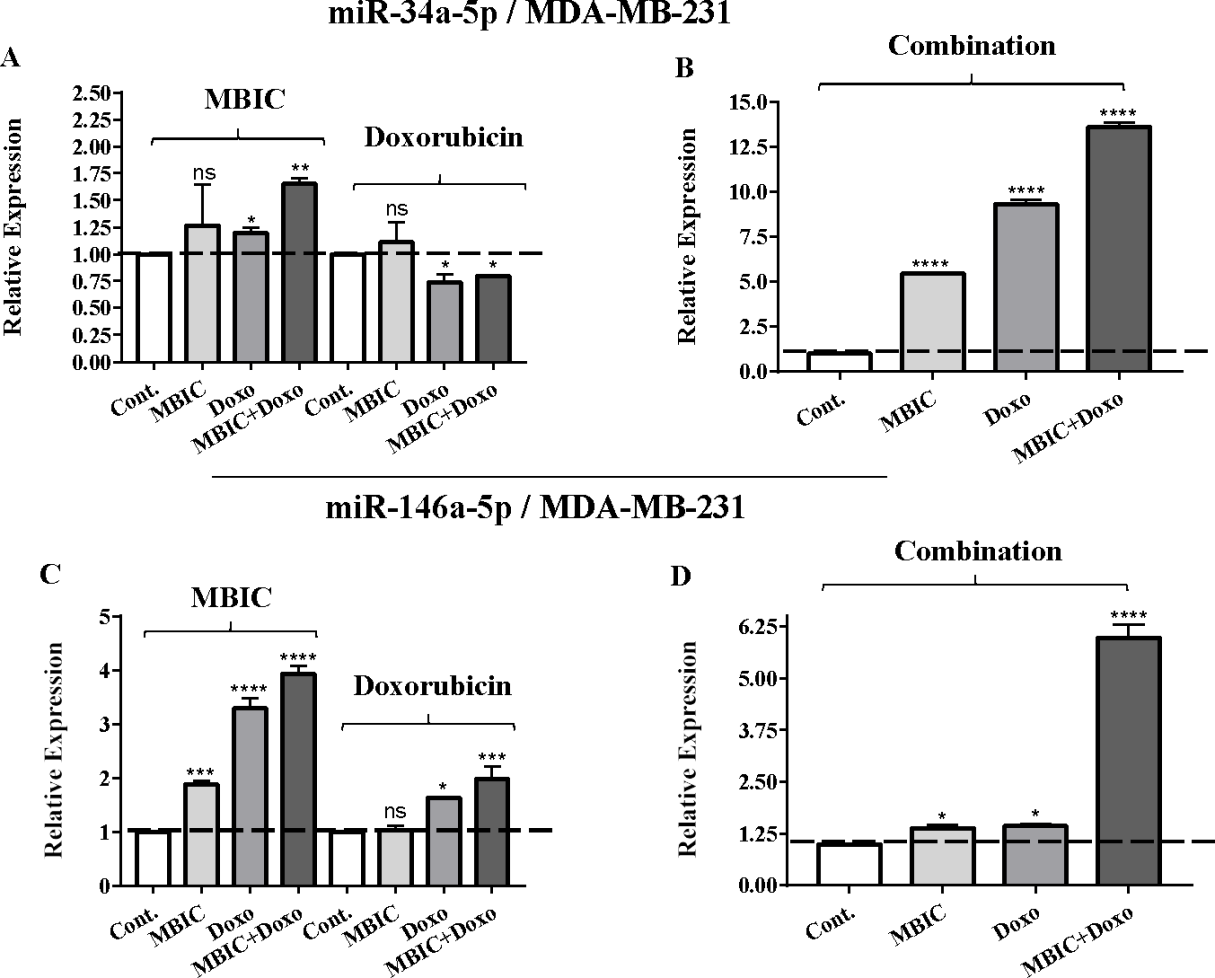
The role of miRNAs (miRs) miR-34a and miR-146a in synergistic effect of MBIC with doxorubicin in MDA-MB-231 cells. Total RNA was extracted and reverse transcribed with their specific designed primers. The level of miRs were normalized to a small RNA (U6) and compared with this endogenous control (set as one-fold). Individual miRNA profiling was done using qRT-PCR analysis. The expression level of miR-34a (A & B) and miR-146a (C & D) in MDA-MB-231 cells following 24 h treatment with MBIC, doxorubicin (A & C) and combination (B & D) of these two drugs are shown. The bars show the fold change of each treated group compared with untreated group (Cont.) as a horizontal dashed line. * *p* < 0.05. ** *p* < 0.01, *** *p* < 0.001, **** *p* < 0.0001 and “ns” indicates not significant compared with untreated control (Cont.). Results are mean + standard deviation of three independent experiments.

**Figure 4:**
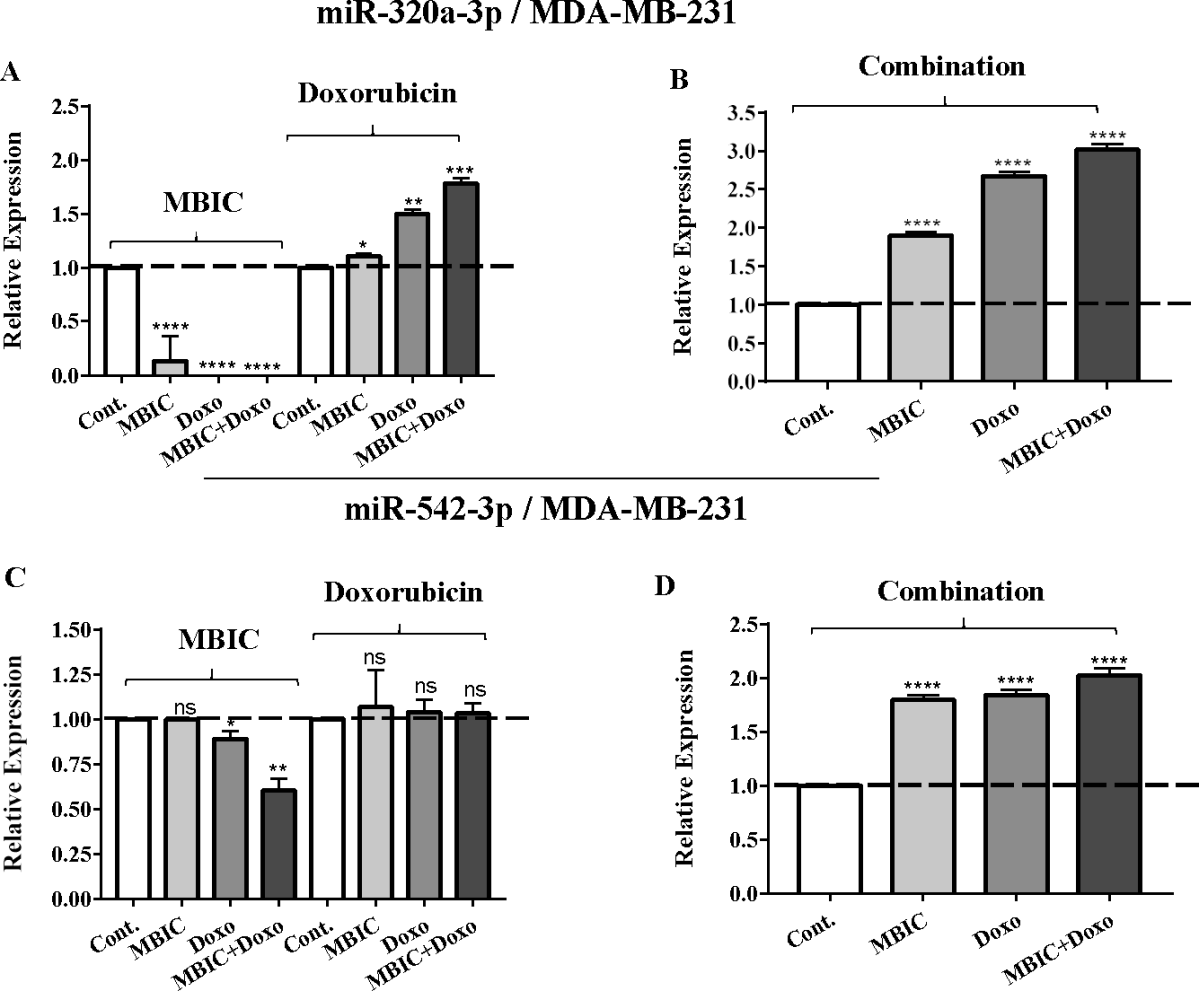
The role of miRNAs (miRs) miR-320a and miR-542 in synergistic effect of MBIC with doxorubicin in MDA-MB-231 cells. Total RNA was extracted and reverse transcribed with their specific designed primers. The level of miRs were normalized to a small RNA (U6) and compared with this endogenous control (set as one-fold). Individual miRNA profiling was done using qRT-PCR analysis. The expression level of miR-320a (A & B) and miR-542a (C & D) in MDA-MB-231 cells following 24 h treatment with MBIC, doxorubicin (A & C) and combination (B & D) of these two drugs are shown. The bars show the fold change of each treated group compared with untreated group (Cont.) as a horizontal dashed line. * *p* < 0.05. ** *p* < 0.01, *** *p* < 0.001, **** *p* < 0.0001 and “ns” indicates not significant compared with untreated control (Cont.). Results are mean + standard deviation of three independent experiments.

As the greatest synergistic anticancer effect was observed following the combined treatment of MBIC with doxorubicin in MDA-MB-231 and MCF-7 cell lines, further the effect of both drugs on the expression of several miRNAs including miR-34a, miR-146a, miR-320a and miR-542 were determined (Figs. 1A–1D, 2A–2D, 3A–3D & 4A–4D; Tables 4 &  5). MBIC treatment at 2 × IC_50_ concentration caused 9.5-fold elevated expression level of miR-34a in MCF-7 cells, but only 1.7-fold increase in MDA-MB-231 cells. The elevated level of expression of miR-34a was 1.5-fold in doxorubicin-treated MCF-7, while the result in doxorubicin treatment of MDA-MB-231 was reduced 0.8-fold. Following the combined drug treatment, a marked increase of 32.3-fold in expression level of miR-34a in MCF-7, and 13.9-fold in expression level of miR-34a in MDA-MB-231 cells were observed.

**Table 4:** Fold differences of miRNAs (miRs) after application of MBIC, doxorubicin in single or combination treatment in MCF-7 cells. Fold differences for the expression of four miRNAs are shown; miR-34a, miR-146a, miR-320a and miR-542 in three groups of treatment; MBIC, doxorubicin and combination treatments compared with untreated (2^−ΔΔCT^) in MCF-7 human breast cancer cells.

MCF-7	Fold differences relative to untreated ( 2^−ΔΔCT^)											
	MBIC				Doxorubicin				Combination			
Target microRNA	Cont.	$ \frac{1}{2} $ × IC_50_	IC_50_	2 × IC_50_	Cont.	$ \frac{1}{2} $ × IC_50_	IC_50_	2 × IC_50_	Cont.	$ \frac{1}{2} $ × IC_50_	IC_50_	2 × IC_50_
miR-34a	1.0	1.0 ± 0.0	8.2 ± 0.0	9.5 ± 0.2	1.0	1.2 ± 0.0	1.3 ± 0.0	1.5 ± 0.0	1.0	1.6 ± 0.0	15.1 ± 0.9	32.3 ± 1.2
miR-146a	1.0	0.3 ± 0.2	0.2 ± 0.1	0.5 ± 0.0	1.0	1.0 ± 0.0	0.7 ± 0.1	0.8 ± 0.1	1.0	0.5 ± 0.1	0.4 ± 0.0	0.2 ± 0.0
miR-320a	1.0	1.2 ± 0.1	5.4 ± 0.3	4.6 ± 0.3	1.0	0.9 ± 0.0	1.0 ± 0.0	1.6 ± 0.0	1.0	2.4 ± 0.0	16.7 ± 1.3	30.9 ± 2.8
miR-542	1.0	0.8 ± 0.2	0.8 ± 0.0	0.9 ± 0.2	1.0	1.1 ± 0.3	1.5 ± 0.1	1.5 ± 0.2	1.0	1.0 ± 0.3	3.0 ± 0.4	3.5 ± 0.2

**Table 5:** Fold differences of miRNAs (miRs) after application of MBIC, doxorubicin in single or combination treatment in MDA-MB-231 cells. Fold differences for the expression of four miRNAs are shown; miR-34a, miR-146a, miR-320a and miR-542 in three groups of treatment; MBIC, doxorubicin and combination treatments compared with untreated (2^−ΔΔCT^) in MDA-MB-231 human breast cancer cells.

MDA-MB-231	Fold differences relative to untreated (2^−ΔΔCT^)											
	MBIC				Doxorubicin				Combination			
Target microRNA	Cont.	$ \frac{1}{2} $ × IC_50_	IC_50_	2 × IC_50_	Cont.	$ \frac{1}{2} $ × IC_50_	IC_50_	2 × IC_50_	Cont.	$ \frac{1}{2} $ × IC_50_	IC_50_	2 × IC_50_
miR-34a	1.0	1.1 ± 0.3	1.2 ± 0.0	1.7 ± 0.0	1.0	0.8 ± 0.1	0.6 ± 0.0	0.8 ± 0.0	1.0	5.5 ± 0.1	9.6 ± 0.3	13.9 ± 0.6
miR-146a	1.0	1.9 ± 0.1	3.5 ± 0.3	4.0 ± 0.2	1.0	1.1 ± 0.1	1.6 ± 0.0	2.0 ± 0.3	1.0	1.4 ± 0.2	1.5 ± 0.1	6.2 ± 0.4
miR-320a	1.0	1.0 ± 0.3	0.5 ± 0.0	0.4 ± 0.0	1.0	1.1 ± 0.1	1.5 ± 0.1	1.8 ± 0.2	1.0	1.9 ± 0.2	2.7 ± 0.2	2.3 ± 0.3
miR-542	1.0	1.0 ± 0.0	0.9 ± 0.1	0.6 ± 0.2	1.0	1.0 ± 0.2	1.0 ± 0.0	1.0 ± 0.0	1.0	1.8 ± 0.0	1.8 ± 0.0	2.1 ± 0.1

Individual treatment with MBIC and doxorubicin at 2 × IC_50_ concentration in MCF-7 cells caused a 0.5-fold and 0.8-fold decrease expression of miR-146a, respectively. In contrast, combined treatment with the two drugs reduced the expression of miR-146a to 0.2-fold in MCF-7 cells. In MDA-MB-231 cells, individual treatment with MBIC and doxorubicin caused 4-fold and 2-fold increase of expression of miR-146a respectively, and followed by an increase of 6.2-fold in the expression of miR-146a in combined treatment of MDA-MB-231 cells.

The expression of miR-320a was elevated by a 30.9-fold following the combined treatment in MCF-7 cells, compared to individual treatments with MBIC (4.6-fold) and doxorubicin (1.6-fold). In MDA-MB-231 cells, treatment with MBIC reduced the expression level of miR-320a by 0.4-fold. Doxorubicin caused a 1.8-fold increase in the expression level of miR-320a in MDA-MB-231 cells. Interestingly, the combination of these two drugs caused a 2.3-fold increase of the miR-320a expression.

MBIC reduced the expression level of miR-542 by 0.9-fold and 0.6-fold in MCF-7 and MDA-MB-231 cells, respectively. However, doxorubicin showed an opposite result, with a 1.5-fold increase of expression in MCF-7 and no change of modification in MDA-MB-231 cells. On the other hand, the seemingly synergistic effect of two drugs caused a 3.5-fold and 2.1-fold elevated level in expression of miR-542 at 2 × IC_50_ concentration in MCF-7 and MDA-MB-231 cells, respectively.

**Figure 5:**
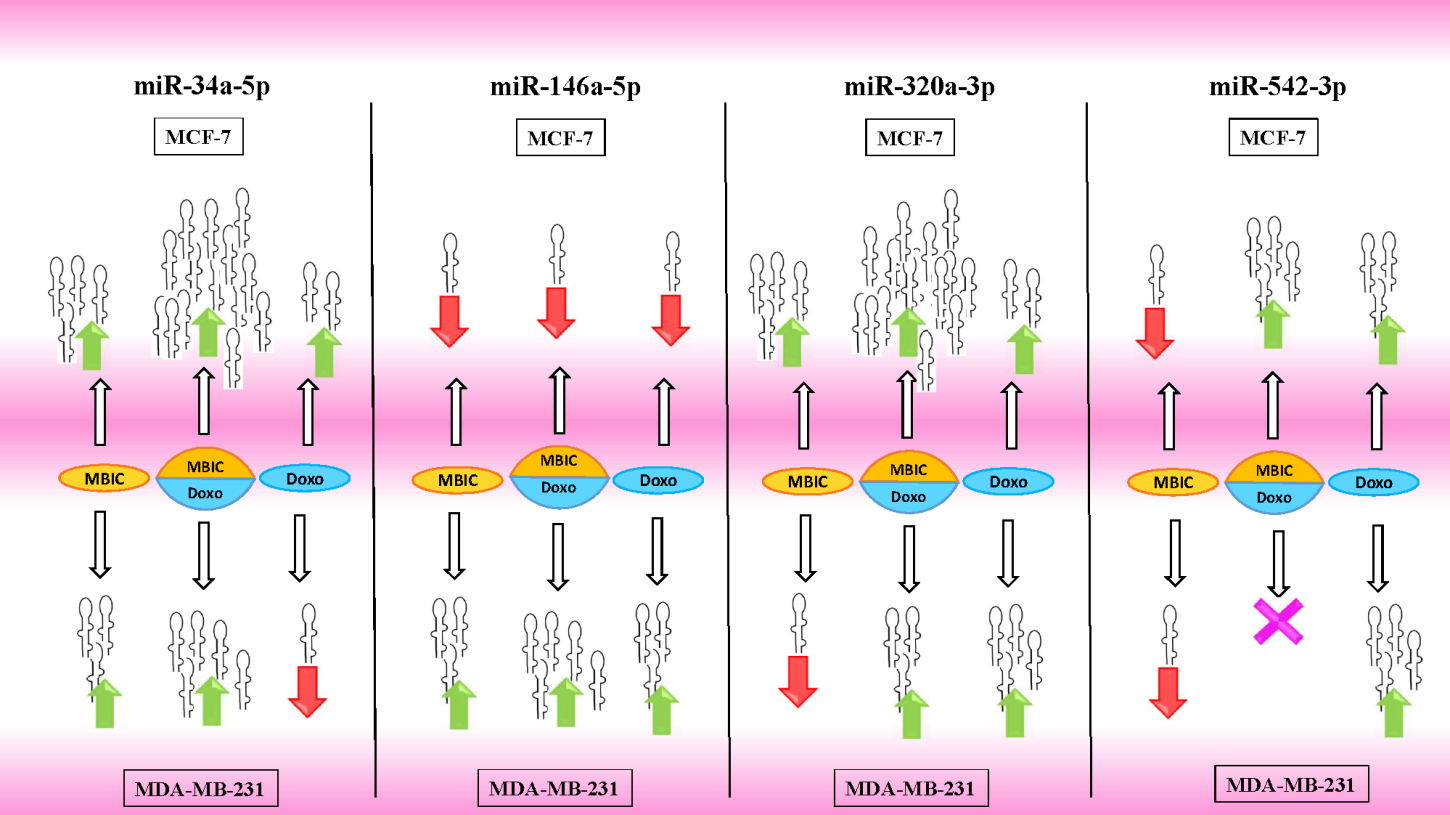
Illustration of elevated or reduced expression level of miRNAs. Four miRNAs including miR-34a, miR-146a, miR-320a and miR-542 in presence or absence of MBIC and doxorubicin in monotherapy or in combination. X shape represents no change in expression level. Up-arrows and down-arrows represent elevation and reduction of expression level respectively.

Tables 4 and  5 showed fold differences for the expression of four miRNAs in three groups of treatment compared to untreated (2^−ΔΔ*CT*^) MCF-7 and MDA-MB-231 cells respectively. In Figs. 1A–1D, 2A–2D, 3A–3D & 4A–4D the bar graphs represented fold-changes in the level of expression of miRNAs. Figure 5 is an illustration of elevation or reduction of expression of miRNAs in breast cancer cell lines.

### Survivin may negatively correlate with miR-34a, miR-320a and miR-542 expression in MCF-7 cell line

**Figure 6:**
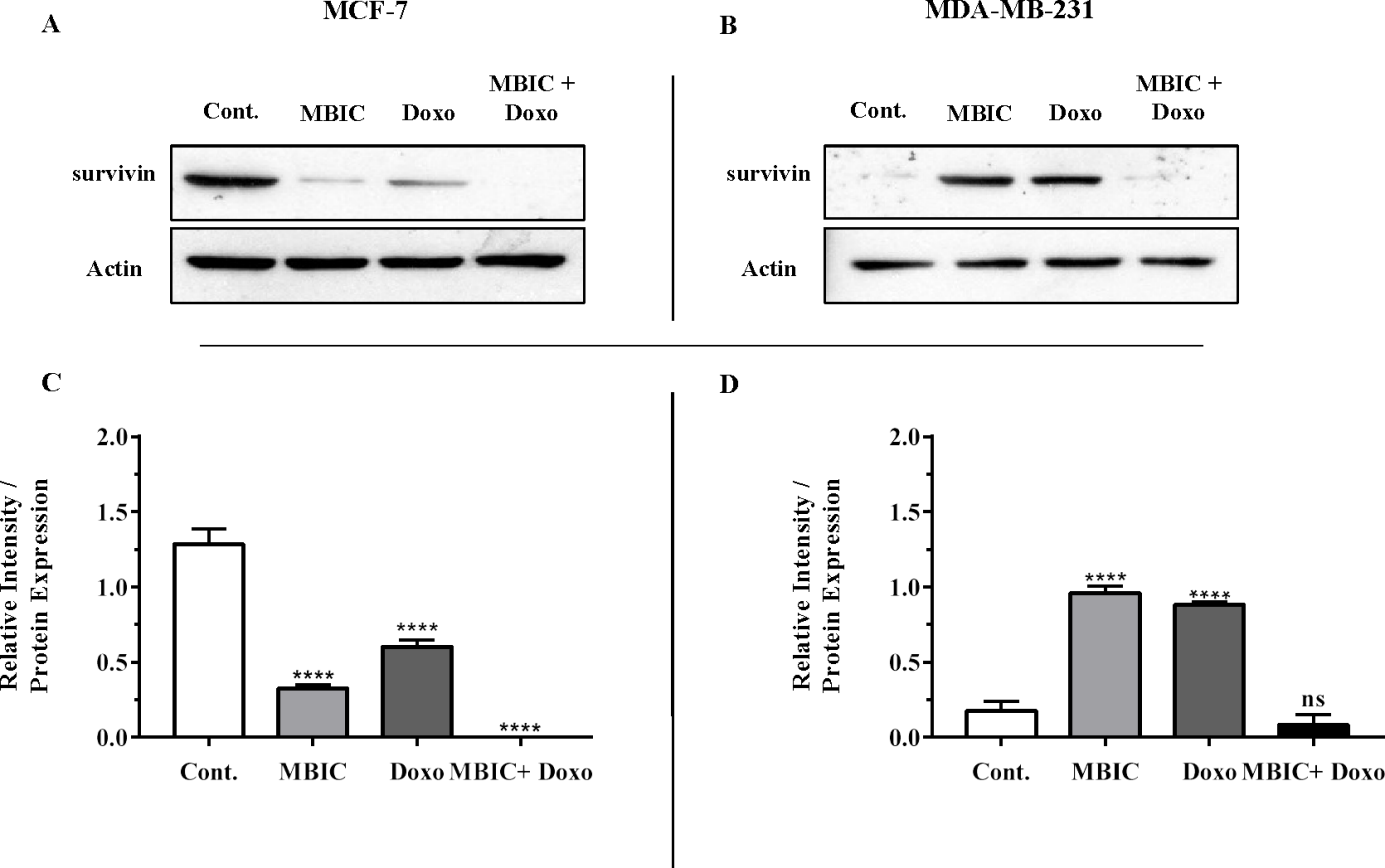
Protein level of survivin following treatment with MBIC and doxorubicin, individually or in combination in MCF-7 and MDA-MB-231 human breast cancer cell lines. (A & B) Representative images from three independent experiments showing western blot analysis to assess the differences in the effect of MBIC, doxorubicin and their combination on the protein level of survivin. MCF-7 (A) and MDA-MB-231 (B) cell lines were treated with MBIC and doxorubicin at 2 × IC_50_ concentration including 1.5 µM of MBIC against MCF-7 cells; 40 µM of MBIC against MDA-MB-231 cells; 11 µM of doxorubicin against MCF-7 cells, and 20 µM of doxorubicin against MDA-MB-231 cells. Selected concentrations in combination therapy were twice the sufficient concentrations of both drugs to cause 50% of cell death. These concentrations were selected to be compared with the expression level of miRNAs at 2 × IC_50_. (C & D) The relative intensity of survivin was normalized with β-actin as internal standard. **** *p* < 0.0001 and “ns” indicate not significant versus untreated control (Cont.).

The protein level of survivin was evaluated in four groups of untreated, MBIC-treated, doxorubicin-treated and combination-treated groups of MDA-MB-231 and MCF-7 cell lines by western blot analysis (Figs. 6A–6D). Comparison between expression level of three miRNAs, miR-34a, miR-320a, miR-542 (Table 4), and the protein level of survivin in the different groups of treatments in the MCF-7 cell line (Figs. 6A &  6C), indicated that there is a negative correlation between the protein level of survivin and these three miRNAs. The observed negative correlation was highlighted in combination treatment of MCF-7 cells, wherein the expression level of miR-34a, miR-320a and miR-542 were elevated 32.3-fold, 30.9-fold and 3.5-fold respectively. Thereby, the three miRNAs at 2 × IC_50_ concentration of combination treatment, may correlate in total suppression of survivin protein in MCF-7 cell-line (Figs. 6A &  6C).

In MBIC-treated MDA-MB-231 cells, the protein level of survivin was elevated compared to untreated cells (Figs. 6B &  6D). This invalidates the negative regulation of survivin protein by miR-34a in MDA-MB-231 cells, as the expression level of miR-34a was elevated following treatment with MBIC (Table 4). MBIC caused a reduction in the expression of miR-542 and miR-320a in treatment of MDA-MB-231 cell line. Therefore, these two miRNAs may be involved in MBIC-induced increase of survivin protein in MDA-MB-231 cells. In doxorubicin-treated MDA-MB-231 cells, increased and stable expression of miR-320a and miR-542, respectively, were not consistent with the negative correlation principal with survivin protein level, as this protein level was increased in doxorubicin-treated MDA-MB-231 cells compared to untreated cells (Figs. 6B & 6D).

Further, combination treatment of MDA-MB-231 cells, caused elevated expression level of miR-34a, miR-320a and miR-542. This elevation did not change the protein level of survivin in comparison with untreated MDA-MB-231 cells. Therefore, the correlation of elevation of miR-34a, miR-320a and miR-542 with survivin protein in combination group of MDA-MB-231, was not significant. Figures 6C & 6D showed the relative intensity of survivin protein expression in each group of treatment in MCF-7 and MDA-MB-231 cell lines.

### NF-κB activation may link to the expression of miR-146a in MDA-MB-231 cell line

**Figure 7:**
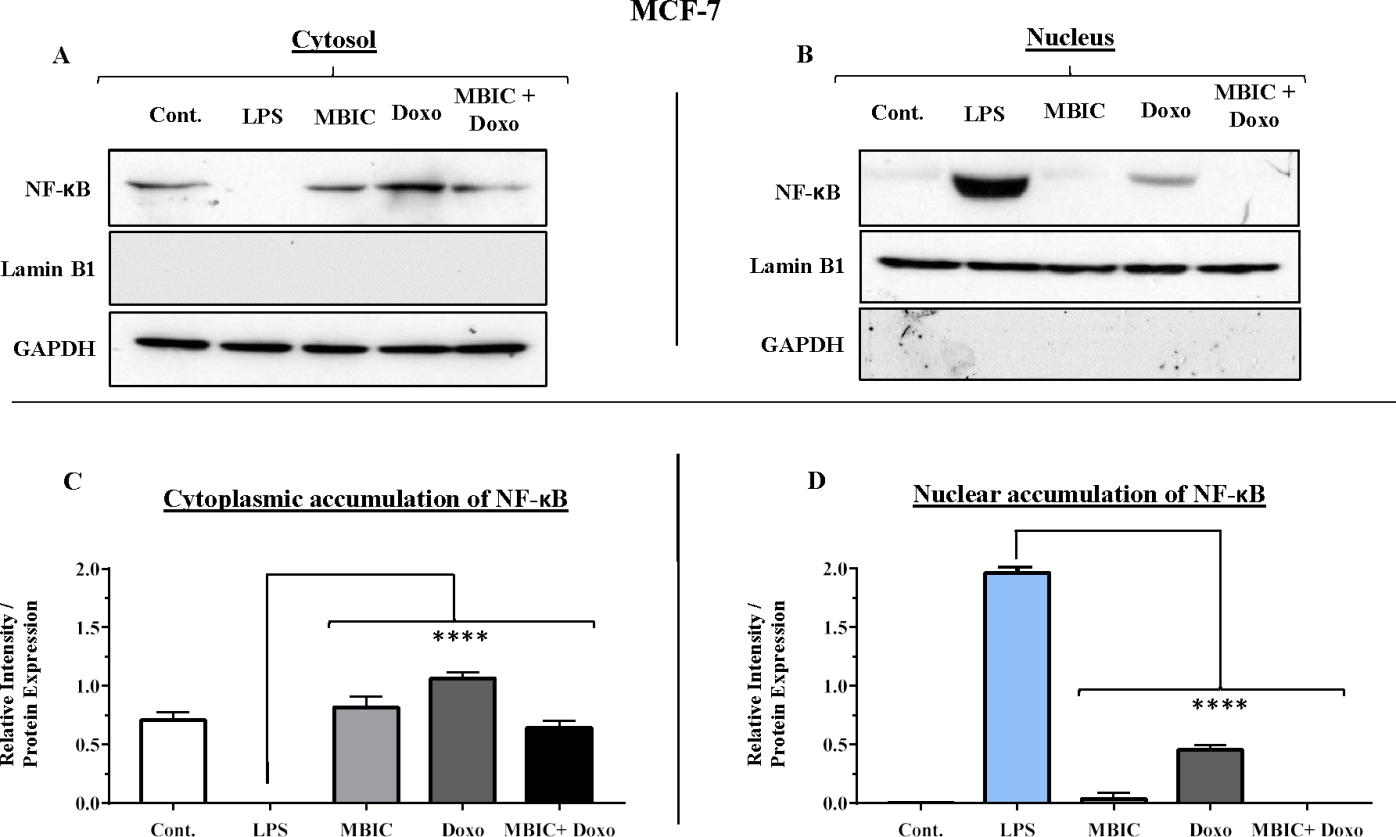
NF-κB activation and its correlation with expression level of miR-146a in MCF-7 cell line. (A & B) Representative images from three independent experiments showing western blot analysis to assess the differences in the effect of MBIC, doxorubicin and their combination on the cytoplasmic (A) and nuclear (B) accumulation of NF-κB. MCF-7 cells were treated with MBIC and doxorubicin at 2 × IC_50_ concentration. 1.5 µM of MBIC and 11 µM of doxorubicin. Selected concentrations in combination therapy were twice the sufficient concentrations of both drugs to cause 50% of cell death. These concentrations were selected to be compared with the expression level of miRNAs at 2 × IC_50_. GAPDH and Lamin B1 proteins were selected as endogenous normalizer reference proteins. These two endogenous normalizers were also probed as negative controls for the opposing fractions (GAPDH for the nuclear and Lamin B1 for the cytosolic fractions). (C & D) The relative intensity of NF-κB was normalized with GAPDH (in cytoplasmic extraction) and Lamin B1 (in nuclear extraction). **** *p* < 0.0001 versus untreated control (Cont.).

**Figure 8:**
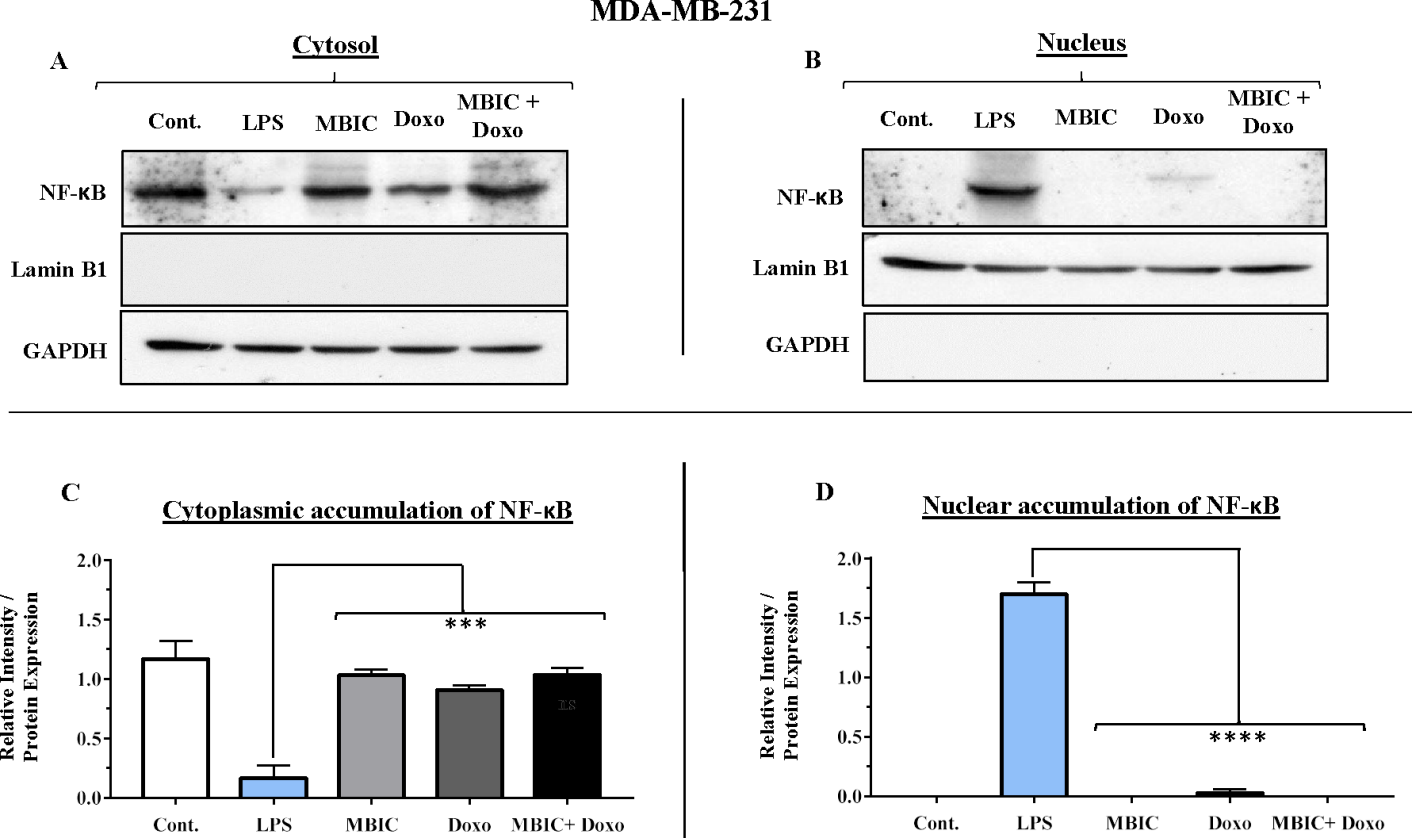
NF-κB activation and its correlation with expression level of miR-146a in MDA-MB-231 cell-line. (A & B) Representative images from three independent experiments showing western blot analysis to assess the differences in the effect of MBIC, doxorubicin and their combination on the cytoplasmic (A) and nuclear (B) accumulation of NF-κB. MDA-MB-231 cells were treated with MBIC and doxorubicin at 2 × IC_50_ concentration. 40 µM of MBIC and 20 µM of doxorubicin. Selected concentrations in combination therapy were twice the sufficient concentrations of both drugs to cause 50% of cell death. These concentrations were selected to be compared with the expression level of miRNAs at 2 × IC_50_. GAPDH and Lamin B1 proteins were selected as endogenous normalizer reference proteins. These two endogenous normalizers were also probed as negative controls for the opposing fractions (GAPDH for the nuclear and Lamin B1 for the cytosolic fractions). (C & D) The relative intensity of NF-κB was normalized with GAPDH (in cytoplasmic extraction) and Lamin B1 (in nuclear extraction). *** *p* < 0.001 and **** *p* < 0.0001 versus untreated control (Cont.).

Since miR-146a at elevated expression level is reported to correlate with suppression of the activity of NF-κB in metastatic human breast cancer cells (Bhaumik et al., 2008), in present study, this correlation was evaluated. Nuclear translocation of NF-κB was evaluated in all four groups of untreated, MBIC-treated, doxorubicin-treated and combination-treated groups in MCF-7 and MDA-MB-231 cell lines (Figs. 7A–7D & 8A–8D). Moreover, nuclear accumulation of NF-κB was evaluated by western blot analysis, and was compared with the expression level of miR-146a. Lipopolysaccharide (LPS), which served as a positive control, stimulated the translocation of NF-κB from cytosol into nucleus in MDA-MB-231 and MCF-7 cells when compared with untreated cells (Figs. 7A–7D & 8A–8D). In Figs. 7C, 7D, 8C and 8D bar graphs showed the relative intensity of NF-κB protein, either inside the cytoplasm or inside the nucleus in MCF-7 and MDA-MB-231 cell lines. In these graphs, the levels of translocation of NF-κB in different treatment groups were compared to the location of NF-κB in LPS-treated group of cells. Injection of LPS assures the translocation of NF-κB. Therefore, it is used as an established model as inducer of NF-κB translocation (Kawai & Akira, 2007).

Table 4 showed down-regulation of miR-146a in all treated groups of MCF-7 cells, while Figs. 7A–7D indicated no nuclear accumulation of NF-κB in MCF-7 cells, especially in MBIC and combination treatment groups, in comparison with LPS-stimulated group of MCF-7 cells. This observation did not support the expected correlation. Since the expression level of miR-146a was reduced under effect of MBIC and doxorubicin, therefore, miR-146a was not sufficient to block the nuclear translocation of NF-κB in MCF-7 cells.

Unlike MCF-7 cells, in MDA-MB-231 the elevated expression level of miR-146a was detected in the MBIC and doxorubicin treatment groups, either individually or in combination. When compared to LPS-treated group, the elevated miR-146a may be involved in the blockage of the nuclear translocation of NF-κB in all treated groups of MDA-MB-231 cells.

In untreated group of cells, both MCF-7 and MDA-MB-231 cell lines exhibited no sign of nuclear translocation of NF-κB. Therefore, if we compare with untreated MCF-7 and MDA-MB-231 cells, elevation of miR-146a may not be involved in inhibition of activation of NF-κB either in MCF-7 or in MDA-MB-231 cell lines.

## Discussion

We previously reported the synergistic reduction of the MDA-MB-231 breast tumor volume in BALB/c nude mice after four weeks of combined treatment with MBIC and doxorubicin. Synergism led to further reduction of 39.5% and 56.8% of tumor volume in multi-drug treatment setting when compared with individual treatments with doxorubicin or MBIC single-drug therapy respectively (Hasanpourghadi et al., 2017). Doxorubicin is a DDA, and despite notable side effects remained as one of the main chemotherapeutic agent widely prescribed for breast cancer (Gavet & Pines, 2010). MBIC is a MTA (Hasanpourghadi et al., 2017) which interferes with the formation of microtubules, perturbing the cytoskeleton to disrupt the movement and translocation of intracellular proteins, such as DNA repair proteins. Combination treatment with MTA and DDA have frequently reported to confer a successful synergism (Blagosklonny, 2007). Due to the disruption of microtubules, there is less chance for damaged DNA to be repaired and this enables the combination therapy to overcome the limitations of single therapy (Blagosklonny, 2007). In this study, the greatest synergistic effect was observed following the combination treatment of MBIC with doxorubicin in MCF-7 and MDA-MB-231 cell lines with CI < 1 (synergism) over the range of 50% to 97% of inhibition. Similarly, the IC _50_value of the combined treatment was much lower than the individual drug therapy with similar level of efficacy. In contrast, treatment with colchicine, nocodazole, paclitaxel and tamoxifen did not exhibit a complete full range of synergistic inhibition over the 50% to 97% range of inhibition of cell proliferation in both breast cancer cell lines.

miRNAs are small noncoding endogenous ∼22 nucleotide-long RNAs which are conserved molecules present in all organisms including plants and animals (Lutter et al., 2010). miRNAs regulate cellular function by inhibiting the translation of protein (translational arrest), and/or by cleaving the messenger RNA (transcript degradation) at post-transcriptional level of protein synthesis (Bhaumik et al., 2008; Rodrigues et al., 2011; Bartel, 2009). Growing evidence revealed that the alterations in the expression of particular miRNAs are involved in cancer development. Several studies reported that miRNAs participate in the epigenetic regulation of intracellular drug disposition and metabolism through altering the expression of drug-targeted proteins (Rodrigues et al., 2011; Pogribny & Beland, 2013; Choudhuri, Cui & Klaassen, 2010). Growing evidence suggested that alteration in the expression of miRNAs, therefore modulation of their biogenesis and function, is an important mechanism underlying the anticancer effect of drugs (Sun et al., 2008). MiRNA are reported to act at the post-transcriptional stage of gene expression, by regulating of the expression of those proteins that are the target of the anticancer drugs (Rodrigues et al., 2011). Overall miRNAs are reported to control the activity of over 50% of all protein-coding genes (Krol, Loedige & Filipowicz, 2010), including a widespread type of protein-protein and protein-RNA interaction. Further, the changes in the expression level of miRNAs have been associated with many human pathologies (Krol, Loedige & Filipowicz, 2010). More recently, there have been increasing reports of the role of miRNAs in the growing resistance to anticancer drugs (Zheng et al., 2010). Considering the data that suggest miRNAs govern cellular fate, miRNA-based investigations may be helpful to understand the successful outcome of these synergistic combinations (Richner et al., 2015).

In this study, we investigated the role of several miRNAs including miR-34a, miR-146a, miR-320a and miR-542 in the synergistic anticancer actions of MBIC and doxorubicin drugs on breast tumors. Fig. 5 illustrates the regulation of miR-34a, miR-320a, miR-146a and miR-542 in MCF-7 and MDA-MB-231 cell lines following MBIC and doxorubicin single and multi-therapies. The fold changes of the expression of these four miRNAs were evaluated at the highest concentration (2 × IC_50_) of MBIC and doxorubicin either as monotherapy or in combination therapy. miR-34a is one of the most prominent miRNAs which generally functions as a tumor suppressor (Zhang et al., 2014). miR-34a is down-regulated in a majority of cancer types and alteration of the expression of miR-34a inhibits cellular proliferation and induces apoptosis in cancer cells (Bader, Brown & Winkler, 2010; Li, Ren & Tang, 2014). miR-34a has been reported to be associated with resistance of a MTA, docetaxel in MCF-7 and MDA-MB-231 cell lines (Kastl, Brown & Schofield, 2012). miR-34a also is known to target the post-transcriptional regulation of survivin protein in gastric cancer cells (Cao et al., 2013). Survivin is a member of inhibitor of apoptosis family (Mckenzie & Grossman, 2012). Increase expression of survivin promotes cancer cell proliferation by suppressing the apoptosis of the cancer cells (Church & Talbot, 2012). In the present study, combined treatment of MBIC with doxorubicin increased the expression of miR-34a in both MCF-7 (32.3 fold) and MDA-MB-231 cell lines (13.9 fold). As miR-34a is a well-known target of p53 (Navarro & Lieberman, 2015; Okada et al., 2014; Raver-Shapira et al., 2007), it is tempting to speculate that this may occur due to enhanced p53 activation as a consequences of DNA damage.

In contrast, increased expression of miR-320a is reported to be linked to suppression of survivin and induction of apoptosis (Diakos et al., 2010). In the present study, the combination therapy of MBIC with doxorubicin resulted in a 30.9-fold and 2.3-fold increase of expression of miR-320a in MCF-7 and MDA-MB-231 cells, respectively.

Several studies reported the increased expression of miR-542 inhibited the expression of survivin in cancer cells (Yoon et al., 2010). The combined treatment of MBIC with doxorubicin treatment was associated with less than four-fold increase in the expression of miR-542 in both cancer cell lines. miR-34a, miR-320a and miR-542 are reported to be inversely correlated with the protein and gene expression levels of survivin in several cancer types (Cao et al., 2013; Diakos et al., 2010; Yoon et al., 2010). Treatment of MCF-7 cells with MBIC, doxorubicin or the drug combination induced up-regulation of miR-34a, miR-320a and miR-542 and this may in turn leads to reduction in the protein level of survivin especially in MCF-7 cells.

The present finding suggests the DDA and MTA combination may exert a synergistically greater effect on the miRNAs, especially in MCF-7 cells. In MDA-MB-231 cell line, reduced expression of miR-542 following treatment with MBIC was consistent with increase of survivin protein compared to untreated group of MDA-MB-231 cells. There was no association between changes in expression level of miR-34a, miR-320a and miR-542 with protein level of survivin in combination treatment of MDA-MB-231 cells. It is noteworthy that combined treatment of MBIC with doxorubicin succeeded to suppress the survivin protein in MDA-MB-231 cells, in comparison with monotherapy of MBIC or doxorubicin individually.

A wide range of stimuli causes nuclear accumulation of the transcription factor, NF-κB (Wan & Lenardo, 2010). Nuclear activation of NF-κB is reported to be associated with increased metastatic potentials (Bhaumik et al., 2008). Highly expressed miR-146a is reported to correlate with suppression of the activity of NF-κB in metastatic human breast cancer cells (Bhaumik et al., 2008). miR-146a in highly expressed condition (by lentivirus), is reported to reduce the metastatic activity of MDA-MB-231 cells (Bhaumik et al., 2008). In this study, the association of nuclear accumulation of NF-κB with the expression level of miR-146a was investigated. MBIC and doxorubicin either as monotherapy or in combination, reduced the expression of miR-146a in MCF-7 cells. However the drug treatment led to blockage of the nuclear accumulation of NF-κB in MCF-7 cells. This finding indicated that miR-146a may not be involved in the inhibitory effect of MBIC and doxorubicin on the translocation of NF-κB in MCF-7 cells. This suggests that the suppression of NF-κB activity could be due to other signaling pathways in MCF-7 cells.

On the contrary, the combination treatment elevated the expression of miR-146a up to 6.2-fold in MDA-MB-231 cells. The elevated expression of miR-146a in combination treatment is higher than elevated level of this miRNA following the individual treatment with MBIC and doxorubicin. The results obtained from MBIC, doxorubicin and combination treatment of MDA-MB-231 cells, demonstrated that elevated expression level of miR-146a is consistent with inhibition of NF-κB nuclear translocation in comparison with LPS-treated MDA-MB-231 cells, the positive control group. miR-146a may be associated with blockage of the nuclear translocation of NF-κB in highly metastatic MDA-MB-231 cell line. Interference with the nuclear translocation curtails the activity of NF-κB and it is reported to cause the loss of invasion and metastatic properties (Bhaumik et al., 2008).

The mechanistic cellular details of miRNAs involvement in depletion of survivin and inhibition of nuclear translocation of NF-κB are yet to be fully characterized. Results of the present study provided further insights into the role of several miRNAs that are employed by the cancer cells. Our results suggestes that several miRNAs that not only may function as tumor suppressors under effect of MBIC and doxorubicin (based on their possible correlation with depletion of survivin protein), but may also act synergistically on miR-34a, miR-320a and miR-542. These preliminary findings may provide the impetus for further studies to gradually characterize the miRNAs network and crosstalk between them.

## Conclusion

Collectively, our observation revealed that miR-34a, miR-320a and miR-542 expression elevated markedly in breast cancer cell lines following treatment with doxorubicin and MBIC in combination. This elevated expression, in a synergistically regulatory network, may be involved in the depletion of an anti-apoptotic protein survivin in less aggressive human breast cancer cells, MCF-7. The synergistic effect of MBIC and doxorubicin in the more aggressive MDA-MB-231 cells could be correlated with elevated expression of miR-146a. Once miR-146a is sharply expressed, the nuclear translocation of NF-κB is subsequently inhibited in MDA-MB-231 cells compared to positive control group of this cell line.

##  Supplemental Information

10.7717/peerj.5577/supp-1 Supplemental Information 1 Western blot raw data The raw data of original film, including protein development of Lamin B1 from cytosol fractions, GAPDH from nucleus fractions of MCF-7 and MDA-MB-231 cell lines.

10.7717/peerj.5577/supp-2 Supplemental Information 2 34miRNA.MCF7 raw data

10.7717/peerj.5577/supp-3 Supplemental Information 3 146miRNA.MCF7 raw data

10.7717/peerj.5577/supp-4 Supplemental Information 4 320miRNA.MCF7 raw data

10.7717/peerj.5577/supp-5 Supplemental Information 5 542miRNA.MCF7 raw data

10.7717/peerj.5577/supp-6 Supplemental Information 6 Original western blot raw data The raw data of original film, including developed proteins, Lamin B1 from cytosol fractions, GAPDH from nucleus fractions of MCF-7 and MDA-MB-231 cell lines.

10.7717/peerj.5577/supp-7 Supplemental Information 7 34.miRNA.MDA raw data

10.7717/peerj.5577/supp-8 Supplemental Information 8 146.miRNA.MDA raw data

10.7717/peerj.5577/supp-9 Supplemental Information 9 320.miRNA.MDA raw data

10.7717/peerj.5577/supp-10 Supplemental Information 10 542.miRNA.MDA raw data

## Additional Information and Declarations

### Data Availability

The following information was supplied regarding data availability: The raw data are provided in the Supplemental Files.
