# Supplementary material for: The role of miRNAs 34a, 146a, 320a and 542 in the synergistic anticancer effects of methyl 2-(5-fluoro-2-hydroxyphenyl)-1H- benzo[d]imidazole-5-carboxylate (MBIC) with doxorubicin in breast cancer cells
Source: PeerJ. 2018 Sep 17;6:e5577. doi: 10.7717/peerj.5577 (PMC6147144; doi:10.7717/peerj.5577)
Supplement: Supplemental Information 1 — The raw data of original film, including protein development of Lamin B1 from cytosol fractions, GAPDH from nucleus fractions of MCF-7 and MDA-MB-231 cell lines. [file peerj-06-5577-s001.zip › numbers.western blot data.pdf]

1) Survivin. MCF7 Cells
2) Actin. MCF7 Cells
3) Survivin. MDA-MB-231 Cells
4) Actin. MDA-MB-231 Cells

5) NF-kB. MCF7 Cells (Cytosol)
6) GAPDH. MCF7 Cells (Cytosol)
7) NF-kB. MCF7 Cells (Nucleus)
8) Lamin B1. MCF7 Cells (Nucleus)

9) NF-kB. MDA-MB-231 Cells (Cytosol)
10) GAPDH. MDA-MB-231 Cells (Cytosol)
11) NF-kB. MDA-MB-231 Cells (Nucleus)
12) Lamin B1. MDA-MB-231 Cells (Nucelus)
