# Supplementary figures and images for: The role of miRNAs 34a, 146a, 320a and 542 in the synergistic anticancer effects of methyl 2-(5-fluoro-2-hydroxyphenyl)-1H- benzo[d]imidazole-5-carboxylate (MBIC) with doxorubicin in breast cancer cells
Source: PeerJ. 2018 Sep 17;6:e5577. doi: 10.7717/peerj.5577 (PMC6147144; doi:10.7717/peerj.5577)

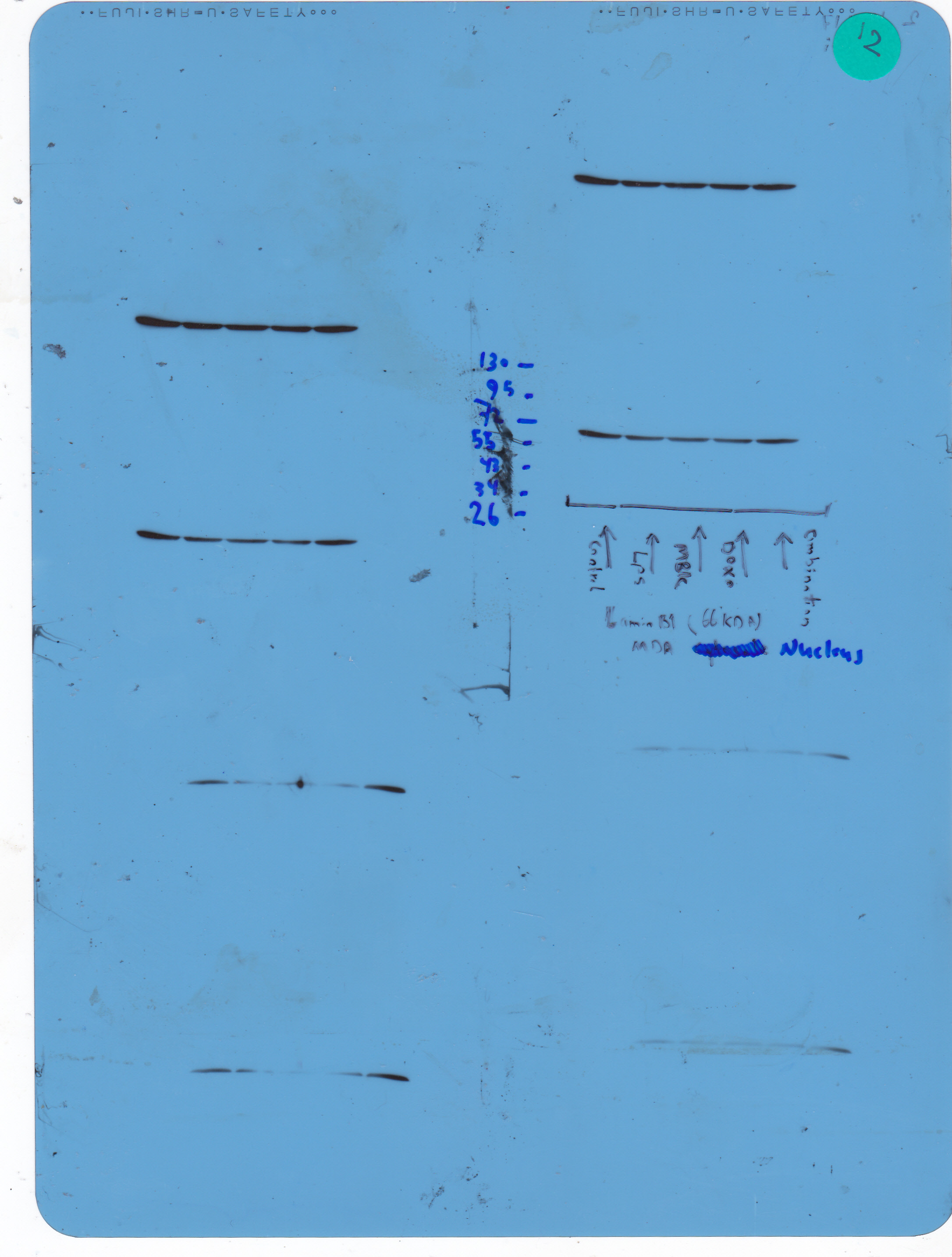

Supplement: Supplemental Information 1 — The raw data of original film, including protein development of Lamin B1 from cytosol fractions, GAPDH from nucleus fractions of MCF-7 and MDA-MB-231 cell lines. [file peerj-06-5577-s001.zip › 12.jpg]

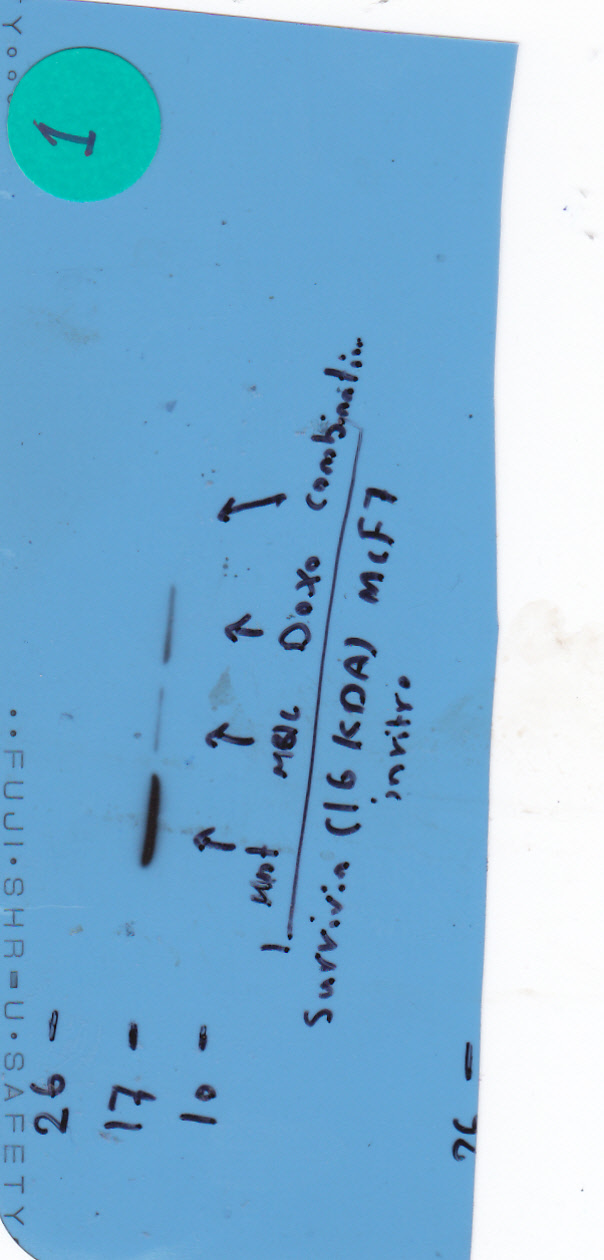

Supplement: Supplemental Information 1 — The raw data of original film, including protein development of Lamin B1 from cytosol fractions, GAPDH from nucleus fractions of MCF-7 and MDA-MB-231 cell lines. [file peerj-06-5577-s001.zip › 1.jpg]

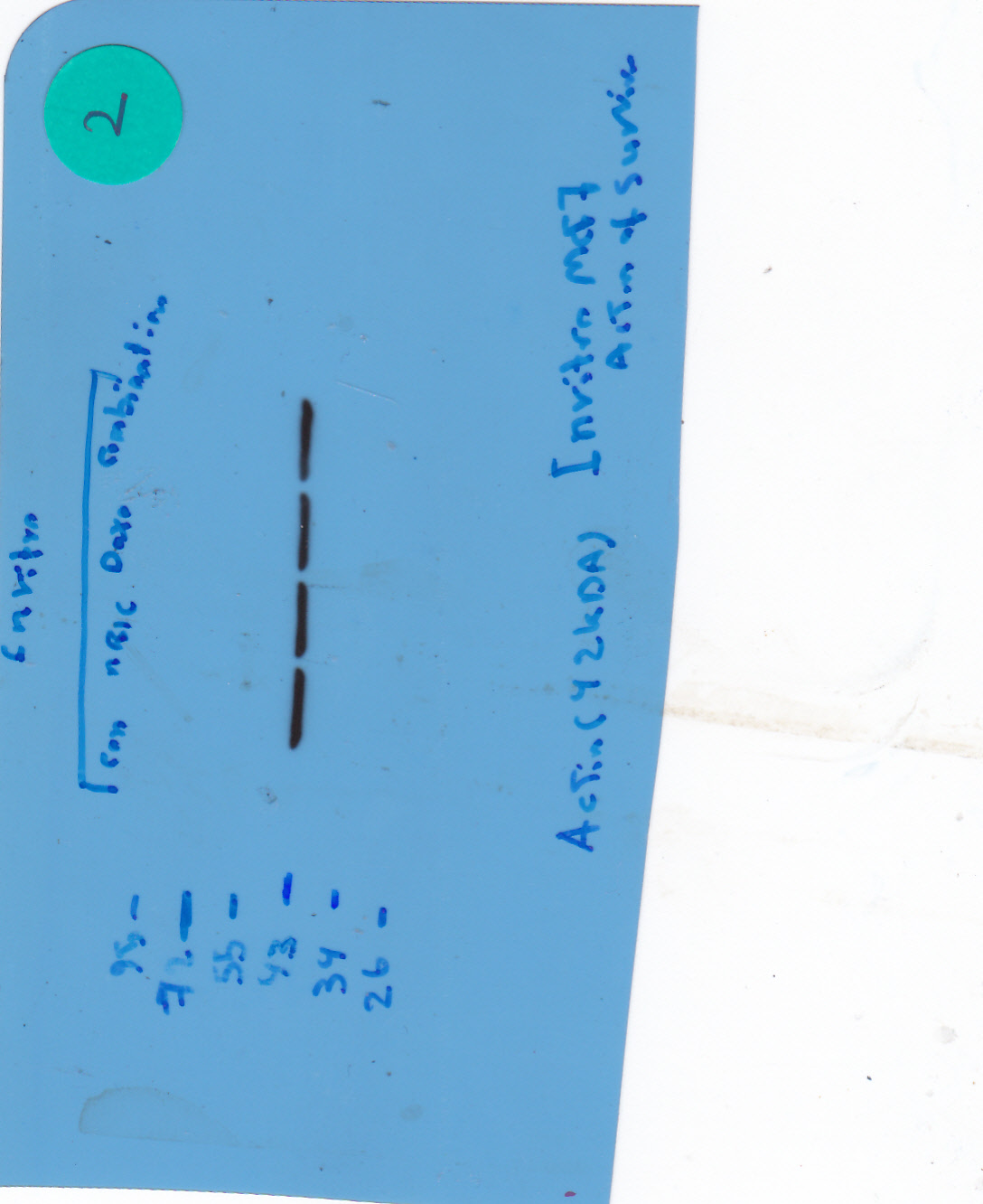

Supplement: Supplemental Information 1 — The raw data of original film, including protein development of Lamin B1 from cytosol fractions, GAPDH from nucleus fractions of MCF-7 and MDA-MB-231 cell lines. [file peerj-06-5577-s001.zip › 2.jpg]

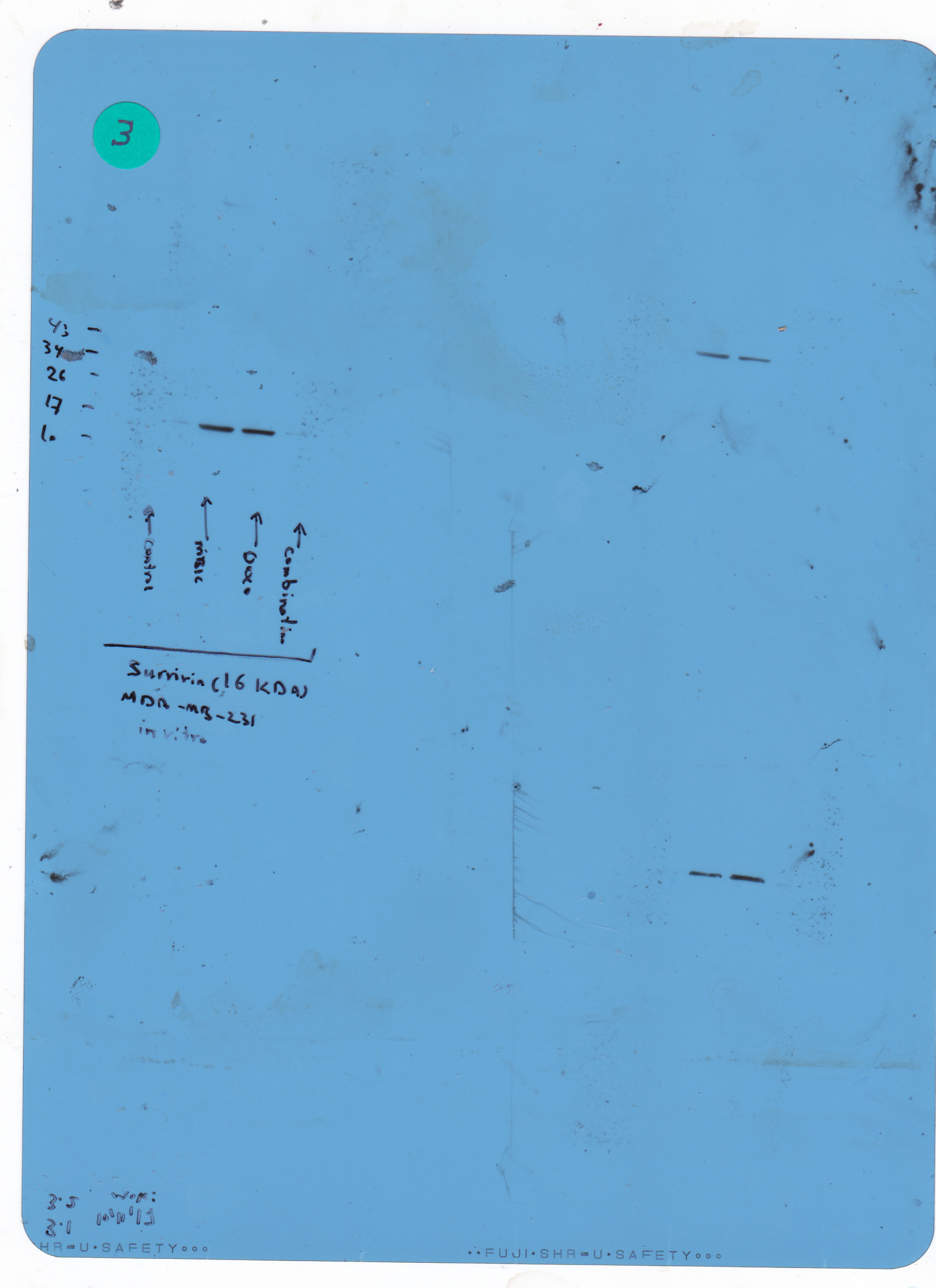

Supplement: Supplemental Information 1 — The raw data of original film, including protein development of Lamin B1 from cytosol fractions, GAPDH from nucleus fractions of MCF-7 and MDA-MB-231 cell lines. [file peerj-06-5577-s001.zip › 3.jpg]

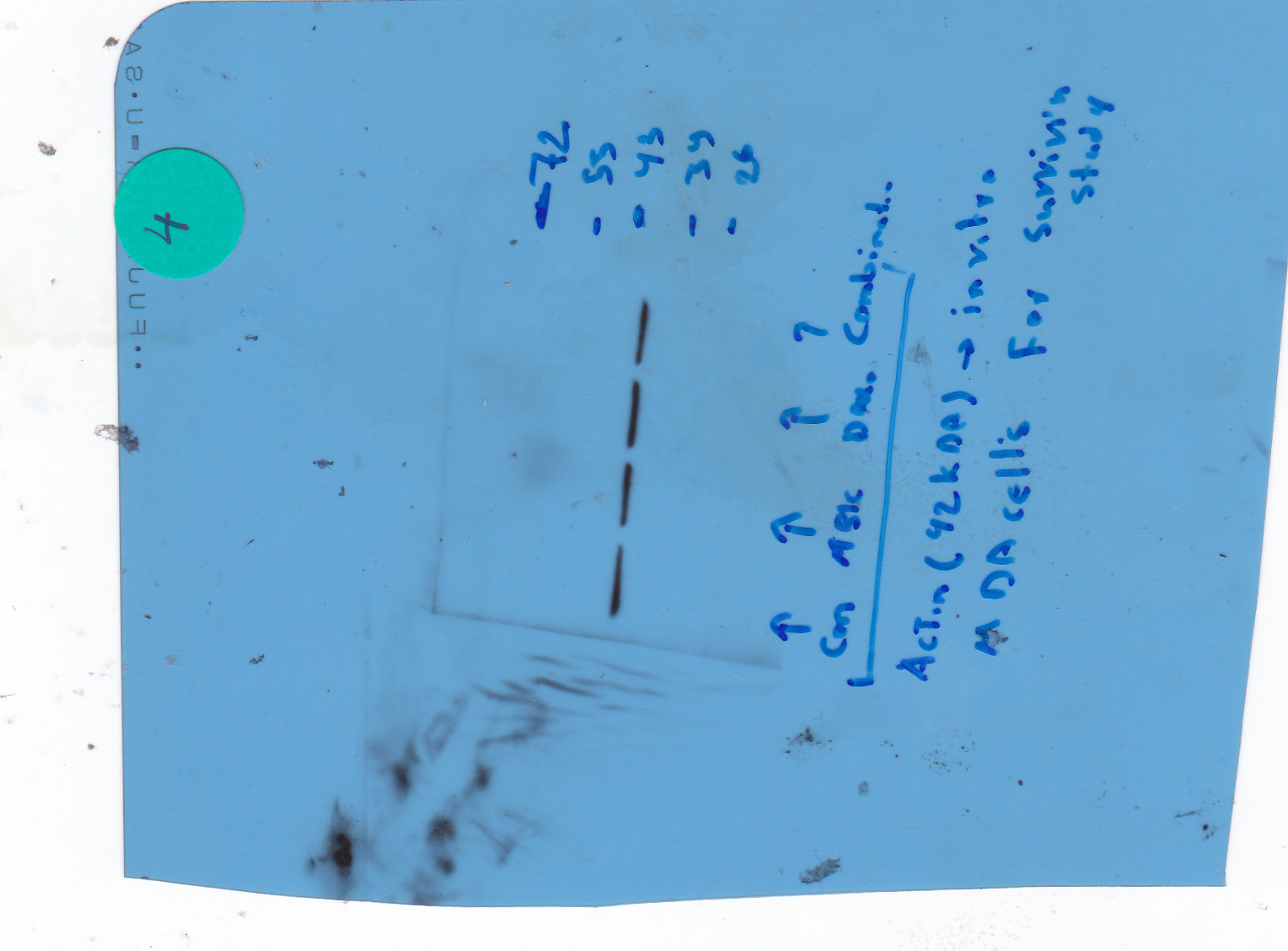

Supplement: Supplemental Information 1 — The raw data of original film, including protein development of Lamin B1 from cytosol fractions, GAPDH from nucleus fractions of MCF-7 and MDA-MB-231 cell lines. [file peerj-06-5577-s001.zip › 4.jpg]

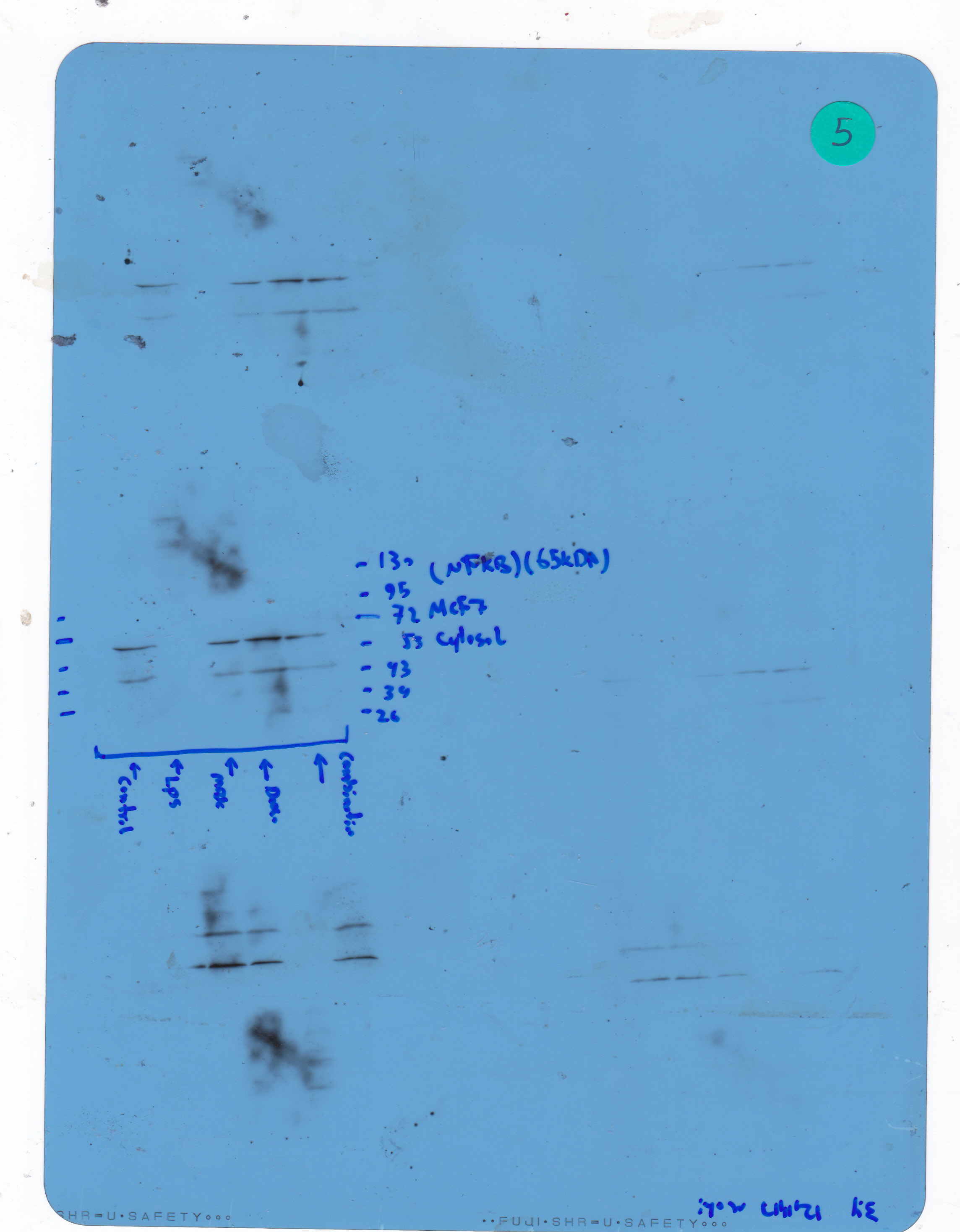

Supplement: Supplemental Information 1 — The raw data of original film, including protein development of Lamin B1 from cytosol fractions, GAPDH from nucleus fractions of MCF-7 and MDA-MB-231 cell lines. [file peerj-06-5577-s001.zip › 5.jpg]

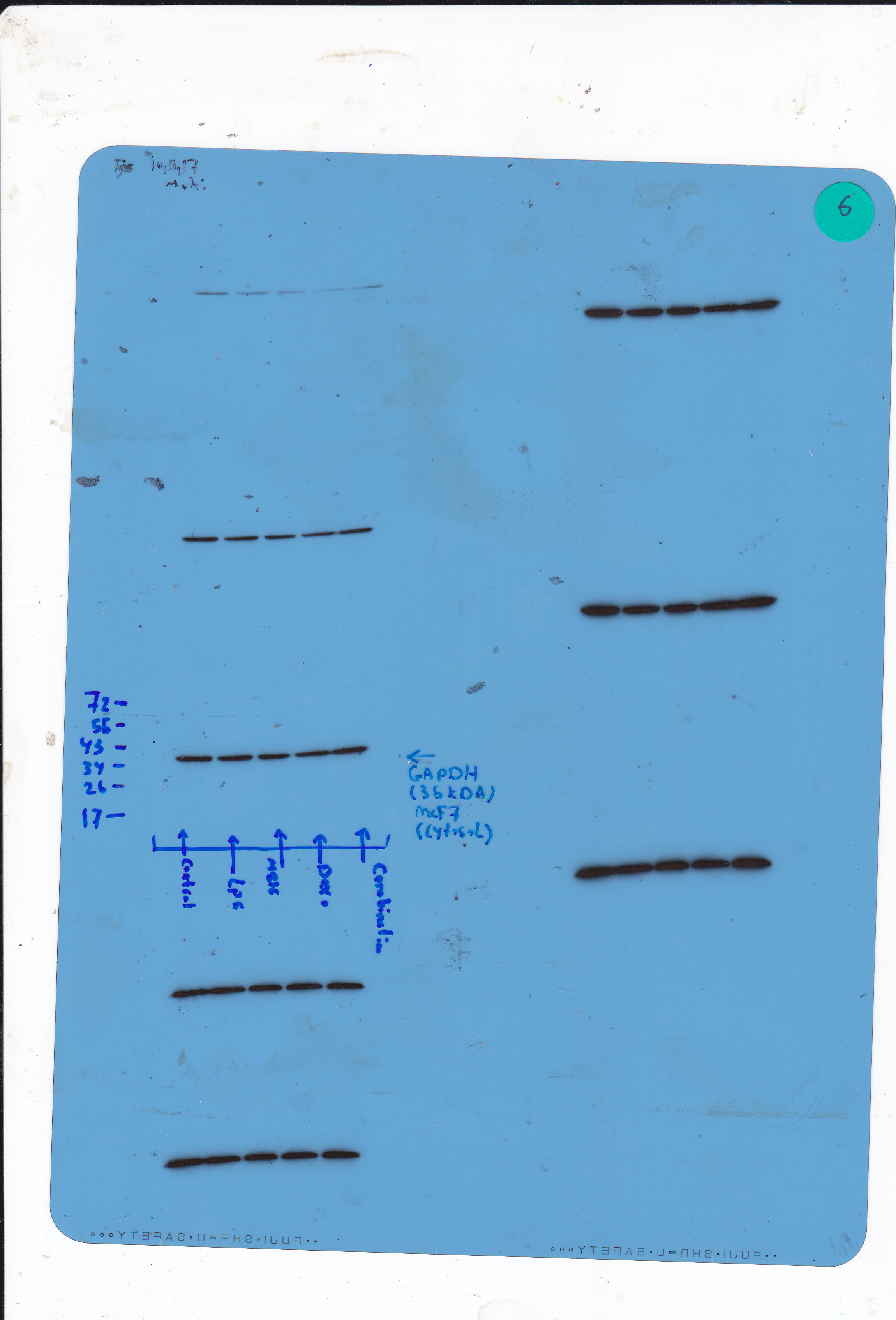

Supplement: Supplemental Information 1 — The raw data of original film, including protein development of Lamin B1 from cytosol fractions, GAPDH from nucleus fractions of MCF-7 and MDA-MB-231 cell lines. [file peerj-06-5577-s001.zip › 6.jpg]

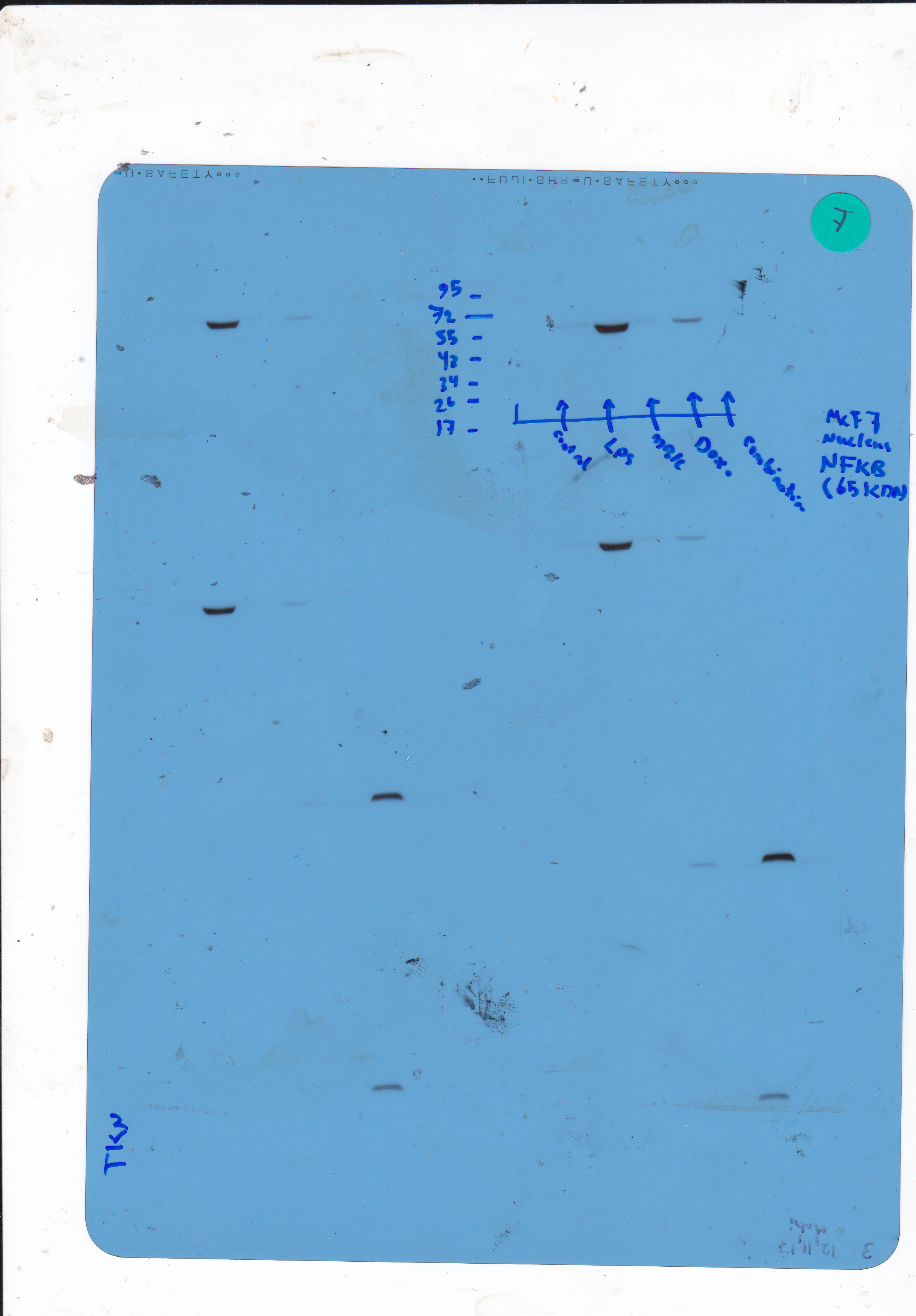

Supplement: Supplemental Information 1 — The raw data of original film, including protein development of Lamin B1 from cytosol fractions, GAPDH from nucleus fractions of MCF-7 and MDA-MB-231 cell lines. [file peerj-06-5577-s001.zip › 7.jpg]

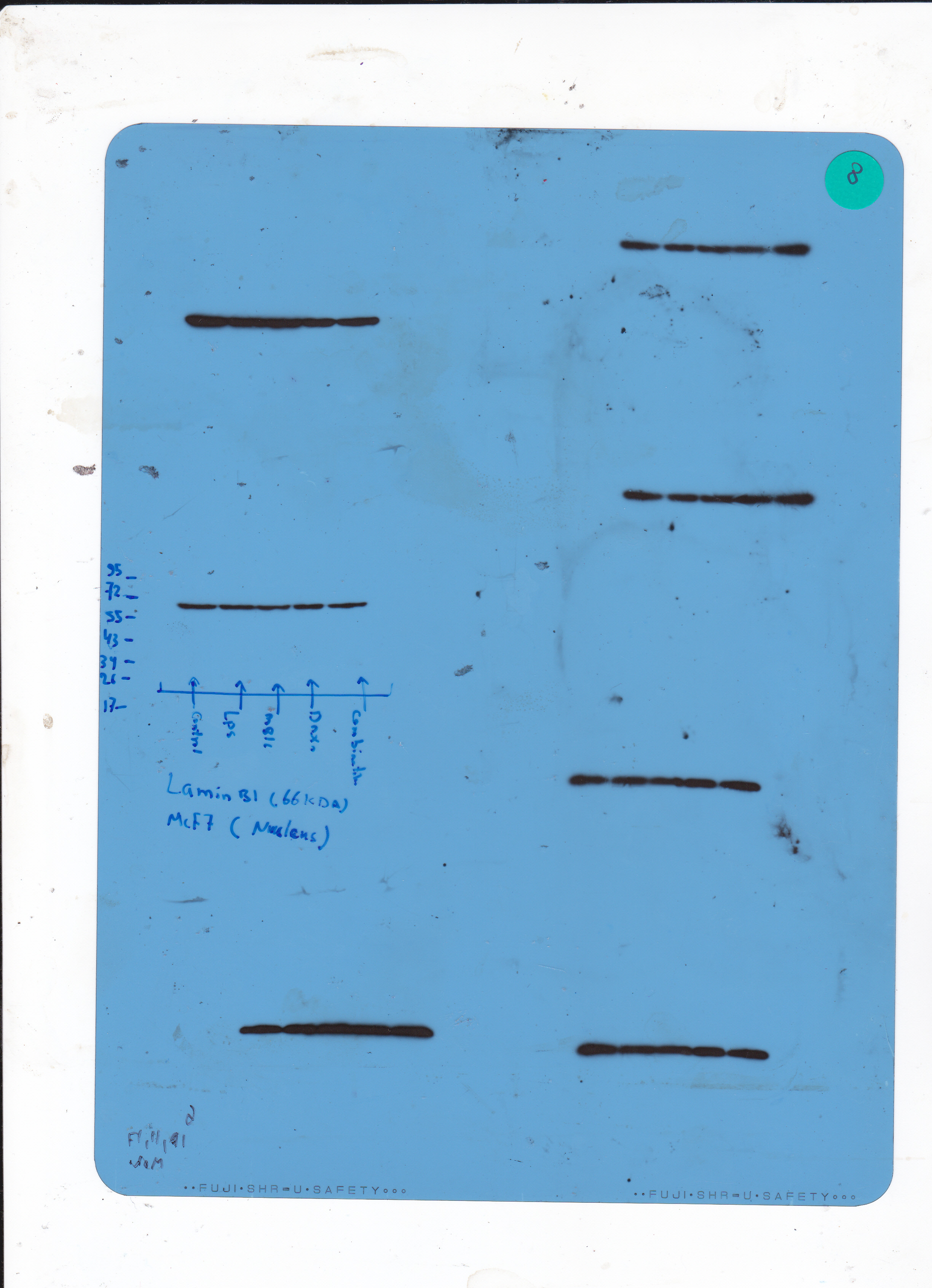

Supplement: Supplemental Information 1 — The raw data of original film, including protein development of Lamin B1 from cytosol fractions, GAPDH from nucleus fractions of MCF-7 and MDA-MB-231 cell lines. [file peerj-06-5577-s001.zip › 8.jpg]

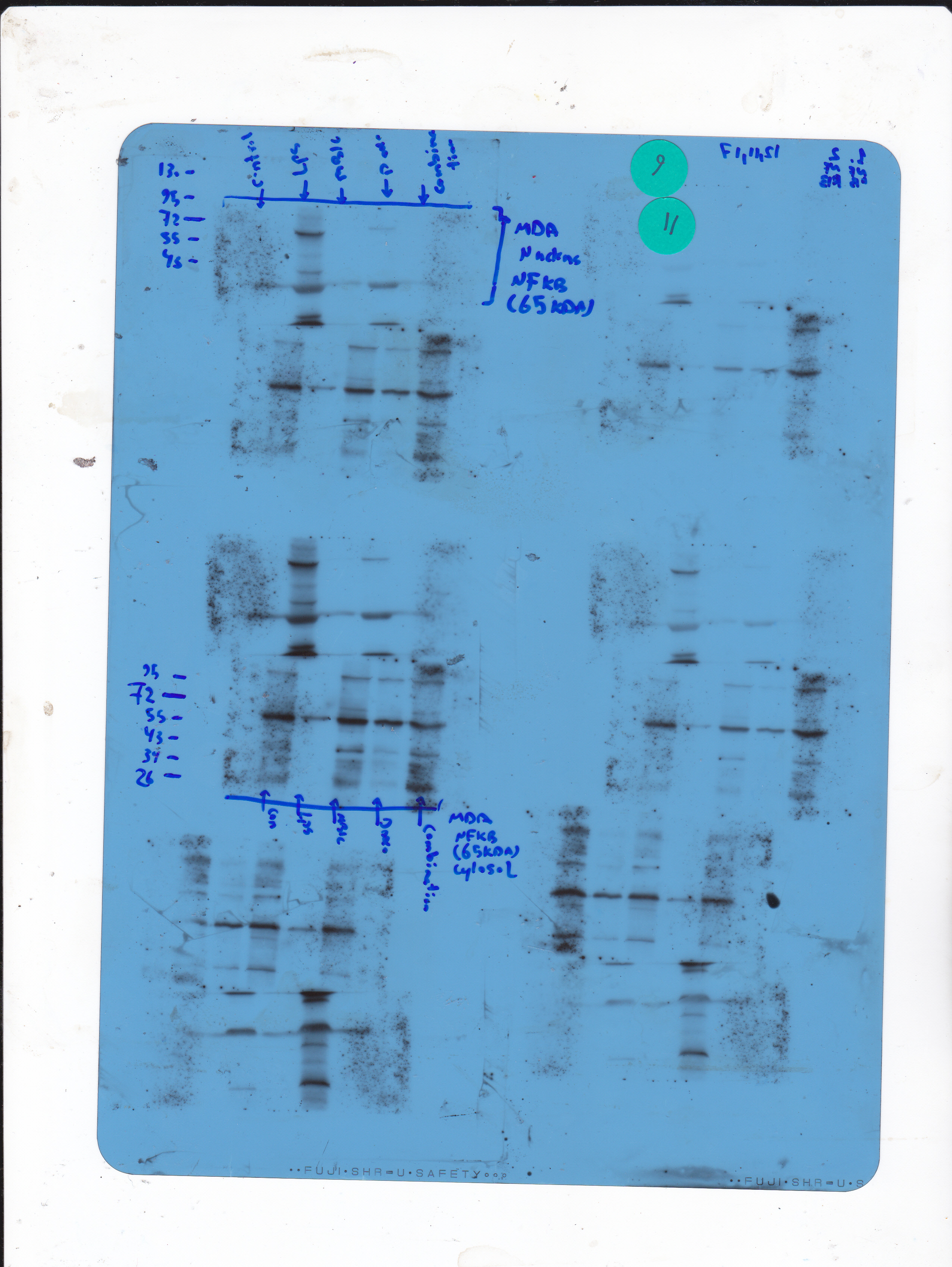

Supplement: Supplemental Information 1 — The raw data of original film, including protein development of Lamin B1 from cytosol fractions, GAPDH from nucleus fractions of MCF-7 and MDA-MB-231 cell lines. [file peerj-06-5577-s001.zip › 9&11.jpg]

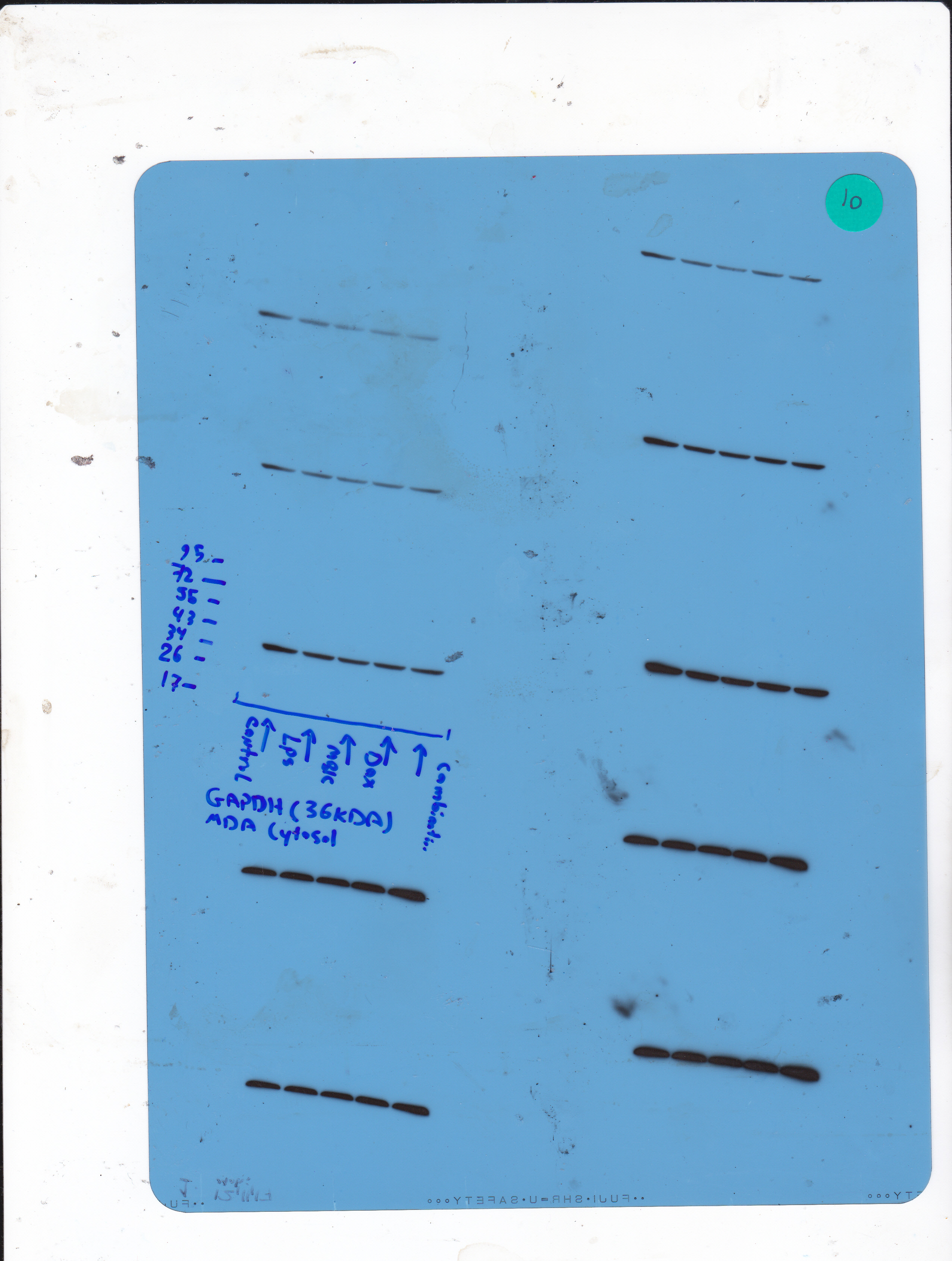

Supplement: Supplemental Information 1 — The raw data of original film, including protein development of Lamin B1 from cytosol fractions, GAPDH from nucleus fractions of MCF-7 and MDA-MB-231 cell lines. [file peerj-06-5577-s001.zip › 10.jpg]
