# Supplementary material for: The role of miRNAs 34a, 146a, 320a and 542 in the synergistic anticancer effects of methyl 2-(5-fluoro-2-hydroxyphenyl)-1H- benzo[d]imidazole-5-carboxylate (MBIC) with doxorubicin in breast cancer cells
Source: PeerJ. 2018 Sep 17;6:e5577. doi: 10.7717/peerj.5577 (PMC6147144; doi:10.7717/peerj.5577)
Supplement: Supplemental Information 6 — The raw data of original film, including developed proteins, Lamin B1 from cytosol fractions, GAPDH from nucleus fractions of MCF-7 and MDA-MB-231 cell lines. [file peerj-06-5577-s006.pdf]

4pt. ~~15 min~~ 8 min
3.4

GAPDH mcf7 Nucleus →

	Con	LPS	MBIC	Doxo	Combo
72 –					
55 –					
43 –					
34 –					
26 –					
17 –					

← GAPDH of Nucleus (MCF7) (Negative Control)

GAPDH MDA-MB Nucleus →

(35.8 KDA)

	Con	LPS	MBIC	Doxo	Combo
55 –					
43 –					
34 –					

← GAPDH of Nucleus (MDA-MB-231) (Negative control)

	Con	LPS	MBIC	Doxo	Combo
	↓	↓	↓	↓	↓
95 –					
72 –					
55 –					
43 –					
34 –					
26 –					
17 –					

LAmin B (66 KDA) mcf7 Cytosol ←

	Con	LPS	MBIC	Doxo	Combo
	↓	↓	↓	↓	↓
95 –					
72 –					
55 –					
43 –					
34 –					
26 –					
17 –					

LAmin B1 (66 KDA) MDA-MB Cells

← Cytosol (Negative control)

••FUJI•SHR=U•SAFETY•••FUJI•SH

up to 16 min
1 25,8,18

Inclues 10 min
12 min
16 min

95 -
72 -
55 -
43 -
34 -
26 -
17 -
10 -

Con LPS MBK Dopa Combo

GAPDH [16 min]
mcf7
(35.8 kDA)
(Nucleus)

- 95
- 72
- 55
- 43
- 34
- 26
- 17
- 10

Con LPS MBK Dopa Combo

GAPDH [16 min]
MDA-MB-231
(Nucleus)

••oo YTEFAS • U = RHS • IJUF ••
••oo YTEFAS •

••FUJI•SHR=U•SAFETY•••

up.to. 16 min

2. 25.6.18

95 –
72 –
55 –
43 –
34 –
26 –
17 –
10 –

Con LPS MBIC LPS Combi

LAmin B1 (66 KDA) [16 min]

mcf7

Cytosol

Con LPS MBIC Doxo Combi

LAminB1 [16 min]

MDA MB

Cytosol

– 95
– 72
– 55
– 43
– 34
– 26
– 17
